# The miR‐19b‐3p‐MAP2K3‐STAT3 feedback loop regulates cell proliferation and invasion in esophageal squamous cell carcinoma

**DOI:** 10.1002/1878-0261.12934

**Published:** 2021-03-14

**Authors:** Ying Zhang, Weiqing Lu, Yelong Chen, Youbin Lin, Xia Yang, Hu Wang, Zhaoyong Liu

**Affiliations:** ^1^ Department of Pathology Sun Yat‐Sen University Cancer Center Guangzhou China; ^2^ Sun Yat‐Sen University Cancer Center State Key Laboratory of Oncology in South China Collaborative Innovation Center for Cancer Medicine Guangzhou China; ^3^ Department of Orthopaedics First Affiliated Hospital of Shantou University Medical College Shantou China

**Keywords:** MAP2K3, STAT3, miR‐19b‐3p, MDM2

## Abstract

Esophageal squamous cell carcinoma (ESCC) is one of the most refractory malignancies worldwide. Mitogen‐activated protein kinase 3 (MAP2K3) has a contradictory role in tumor progression, and the function and expression patterns of MAP2K3 in ESCC remain to be determined. We found that MAP2K3 expression to be downregulated in ESCC, and MAP2K3 downregulation correlated with clinically poor survival. MAP2K3 inhibited ESCC cell proliferation and invasion *in vitro* and *in vivo*. MAP2K3 suppressed STAT3 expression and activation. Mechanistically, MAPSK3 interacted with MDM2 to promote STAT3 degradation via the ubiquitin–proteasome pathway. Furthermore, exosomal miR‐19b‐3p derived from the plasma of patients with ESCC could suppress MAP2K3 expression to promote ESCC tumorigenesis. STAT3 was found to bind to the *MIR19B* promoter and increased the expression of miR‐19b‐3p in ESCC cells. In summary, our results demonstrated that the miR‐19b‐3p‐MAP2K3‐STAT3 feedback loop regulates ESCC tumorigenesis and elucidates the potential of therapeutically targeting this pathway in ESCC.

## Graphical abstract

The regulatory function of MAP2K3 was studied in esophageal squamous cell carcinoma (ESCC) progression. MAP2K3 was found to inhibit cell proliferation and invasion via the EGFR–STAT3 signaling pathway. MAP2K3 suppressed STAT3 expression and activation through proteasomal degradation. miR‐19b‐3p could suppress MAP2K3 expression and could be transcriptional activated by STAT3 in ESCC cells.

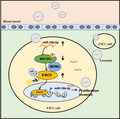

**Abbreviations**
5‐aza: 5‐aza‐2’‐deoxycytidine
CCK‐8: cell counting kit‐8
CHX: cycloheximide
CI: confidence interval
CIS: carcinoma in situ
CRISPR/Cas9: clustered regularly interspaced short palindromic repeats/associated protein 9
DAPI: 4’,6‐diamidino‐2‐phenylindole
DMEM: Dulbecco’s modified Eagle’s medium
EGFR: epidermal growth factor receptor
ESCC: esophageal squamous cell carcinoma
FBS: fetal bovine serum
GO: gene ontology
HR: hazard ratio
IHC: immunohistochemical
IFN: interferon
IP: immunoprecipitation
KO: knockout
MAP2K3: mitogen‐activated protein kinase kinase 3
MAPK: mitogen‐activated protein kinase
MDM2: mouse double minute 2
miRNAs: microRNAs
NC: negative control cells
NSCLC: non‐small‐cell lung cancer
qRT‐PCR: quantitative reverse transcription‐polymerase chain reaction
STAT3: signal transducers and activators of transcription 3
UTRs: 3’ untranslated regions
VEGF: vascular endothelial growth factor

## 1 Introduction

Esophageal cancer, especially esophageal squamous cell carcinoma (ESCC), is one of the most common primary malignant tumors in China, with a 5‐year survival of only 19% [1]. Surgery supplemented by chemoradiotherapy is still the main treatment for esophageal cancer. However, unlike non‐small‐cell lung cancer and melanoma, the most frequent therapeutic targets in ESCC are currently undruggable and novel targets urgently needed [2, 3].

Mitogen‐activated protein kinase 3 (MAP2K3), a dual‐specificity kinase in the MAP kinase kinase (MKK) family, is activated by MKK kinase proteins through Ser‐189 and Thr‐193 phosphorylation. MAP2K3 was identified as an oncogene whose depletion reduces tumor growth and improves biological response to chemotherapy [4, 5]. In ESCC, a MAP2K3 inhibitor was reported to suppress cell growth [6]. However, the role of MAP2K3 in cancer has been challenged recently. A novel loss of MAP2K3 genomic copy number was observed in breast cancer patients and the overexpression of MAP2K3 inhibited breast cancer cell proliferation by promoting cell cycle arrest [7]. Although these findings supported that MAP2K3 plays a crucial role in cancer development, the expression and biological function of MAP2K3 in ESCC remains to be elucidated.

Signal transducer and activator of transcription (STAT) 3, a transcription factor that regulates cell cycle progression, apoptosis, and angiogenesis, belongs to a transcription factor family that transduce cellular signals induced by cytokines and growth factors such as interleukin (IL)‐6 and interferon (IFN) [8]. STAT3 plays an important role in tumorigenesis and its activation induces expression of apoptosis inhibitors and cell cycle regulators that participate in oncogenesis, such as vascular endothelial growth factor (VEGF), cyclin D1, and survivin [9]. STAT3 expression has also been correlated with reduced survival and poor prognosis in patients with ESCC, breast cancer, and lung cancer [10]. However, the correlation between MAP2K3 and STAT3 has never been explored.

Exosomes, which range in size from 30 to 150 nm in diameter, are cell‐derived vesicles and can deliver intracellular cargo, including microRNAs (miRNAs), mRNAs, and proteins to exosome‐recipient cells [11]. miRNAs, as a class of short, noncoding RNAs (~22 nt), could bind to the 3’ untranslated regions (UTRs) of target mRNAs and negatively regulate their target genes [12]. There is growing interest in utilizing exosomes as delivery vehicles for miRNA to contribute to cancer progression and metastasis. For example, cancer cell‐derived exosome transmitted miR‐1910‐3p promotes breast cancer cell proliferation, metastasis, and autophagy [13]. ESCC cell‐derived exosomal miR‐103a‐2‐5p can promote proliferation and migration of tumor cells [14].

In this study, we employed a clustered regularly interspaced short palindromic repeats/associated protein 9 (CRISPR/Cas9) kinome screened and identified MAP2K3 as an ESCC tumor suppressor gene. The expression and function of MAP2K3 and its downstream signaling pathway EGFR/STAT3 were subsequently explored in ESCC. Furthermore, we also found that MAP2K3 is suppressed by miR‐19b‐3p, which is transcriptionally activated by STAT3 in ESCC cells. Our study is the first to demonstrate that the miR‐19b‐3p/MAP2K3/STAT3 feedback loop regulates ESCC tumorigenesis. Therefore, MAP2K3 may represent a predictor and potential therapeutic target for ESCC.

## 2 Material and methods

### 2.1 Cells and patient samples

ESCC cell lines KYSE150, KYSE520, TE1, KYSE180, KYSE450, KYSE410, and the immortalized esophageal epithelial cell line NE1 were maintained in Dulbecco’s modified Eagle’s medium (DMEM, Gibco, NY, USA) and RPMI 1640 supplemented with 10% fetal bovine serum (FBS, Gibco) with 5% CO_2_ in 37 ℃. This study included 140 randomly selected patients with primary ESCC who underwent radical esophageal resection at the First Affiliated Hospital of Shantou University Medical College from 2008 to 2015. 106/140 (75%) were males and 34/140 (25%) were females. The median age was 57.8 years (range, 37–75 years). Plasma samples of ESCC patients (*n* = 7) and the healthy controls (*n* = 7) were obtained from First Affiliated Hospital of Shantou University Medical College. This study was approved by the ethical review committees of First Affiliated Hospital of Shantou University Medical College. Our study methodologies conformed to the standards set by the Declaration of Helsinki. All participants involved in our study provided written informed consent.

### 2.2 Pooled CRISPR/Cas9 sgRNA screen

A pooled lentivirus‐based plasmid library, encompassing 2925 different sgRNAs against 976 human kinase genes (3 sgRNAs per gene, Table S1), was obtained from EdiGene (Beijing, China). The library was cotransfected, with a lentivirus expression system, using Fugene HD (Promega, WI, USA). Lentivirus was packaged and concentrated by using a three‐plasmid system (pCMVR8.74, pcmv‐vsv‐g, and cas9‐2a‐mchy‐bsd) to obtain the high‐titer lentivirus wrapped in cas9‐2a‐mchy‐bsd. Forty‐eight hours after transfection, lentivirus‐containing supernatants were collected and clarified by centrifugation. After selection with puromycin for 7 days, ~2 × 10^8^ cells were infected (MOI of 0.3) and then incubated for 2–3 weeks with or without drug treatment. Genomic DNA was extracted from transfected or control cells, and sgRNA sequences were amplified and subjected to next‐generation sequencing using an Illumina HiSeq 2500 platform.

### 2.3 Nuclear and cytoplasmic extraction and western blot analysis

NE‐PER^TM^ nuclear and cytoplasmic extraction reagents (Thermo Scientific, MA, USA) were used to separate and prepare cytoplasmic and nuclear extracts from ESCC cells. Western blotting was performed as described previously [15]. Antibodies against human β‐actin, MAP2K3, p‐MAP2K3, STAT1, p‐STAT1, EGFR, p‐EGFR, caspase 3, cleaved(cl‐) caspase 3, GFP, Ki67, Flag, STAT3, p‐STAT3, ubiquitin (Ub), HA, MDM2, cleaved(cl‐) PARP, CD9, CD63, and TSG101 were purchased from Cell Signaling Technology (MA, USA).

### 2.4 Immunohistochemistry (IHC) and Immunocytochemistry

IHC staining was performed using the EnVision System using peroxidase labeling (Dako, Carpinteria, CA) as described previously [15]. The expression of MAP2K3, STAT3, or indicated proteins was analyzed based on the proportion and the intensity of positively stained cells. For the intensity of stained cells, four levels were graded as follows: 0: negative; 1: weak; 2: mild; and 3: strong. For the proportion of stained cells, five levels were graded as follows: 0: no staining; 1: positive staining in 1–25% of tumor cells; 2: 26% to 50%; 3: 51% to 75%; 4: >76%. Then, the cell scores were multiplied, and low expression was scores of 0–6, and the expression was high when the score was 8–12. The Immunocytochemistry was performed as described previously [15].

### 2.5 Chemicals, plasmid transfection, and stable cell lines established

Cycloheximide (CHX) and 5‐aza‐2’‐deoxycytidine (5‐aza) were purchased from MCE (NJ, USA). All drugs were dissolved in dimethyl sulfoxide (DMSO; Sigma, MO, USA). The Flag‐wild‐type (WT) MAP2K3 plasmid, dominant‐negative MAP2K3 (S‐A), constituted activated MAP2K3(S‐E) constructed by replacing Ser‐189 and Thr‐193 with Ala or Glu residues, Flag‐MDM2, MDM2^C464A^, GFP‐STAT3 WT, STAT3 (Y705A), STAT3 (S727A), STAT3 ΔN‐terminal domain (NTD), Δ coiled‐coil domain (CCD), ΔDNA‐binding domain (DBD), Δ Linker domain (LD), Δ Src Homology 2 (SH2), Δ Transactivation domain (TAD) plasmids, MIR19B transcriptional activity reporter plasmids, and three mutant type plasmids (mt1: ‐1462/‐1452; mt2: ‐1295/‐1285; and mt3: both mutant) were purchased from Vigene (Guangzhou, China). Plasmid transfection was performed using Lipofectamine 3000 reagent (Invitrogen, NY, USA) according to the manufacturer’s instructions.

The miR‐19b‐3p mimic or inhibitor, and siRNAs were purchased from GenePharma (Jiangsu, China). Transfection of these RNAs was performed using Lipofectamine RNAiMAX (Invitrogen) according to the manufacturer’s instructions. For the stable cell lines, MAP2K3 knockout (KO) cell lines were established using the CRISPR/Cas9 system. Briefly, sgRNA targeting the MAP2K3 gene was designed using the CRISPR Design Online Tool (https://www.genscript.com/gRNA‐design‐tool.html). After transfection, the puromycin‐resistant cells were selected and expanded. Western blot and Sanger sequencing were performed to confirm the knockout. The stable miR‐19b‐3p expression cell lines were established though infection ESCC cells with lentivirus particles according to the manufacturer's instructions. Real‐time PCR was used to investigate the level of mRNA expression after lentivirus infection.

### 2.6 Invasion assay

Transwell invasion assay was performed to detect the ESCC cell invasion ability. Briefly, 5 × 10^5^ ESCC cells in serum‐free medium (Gibco) were placed on the Matrigel (BD, USA)‐coated membrane in the Transwell chamber (Corning Costar Corp) and the medium with FBS was added in the lower chamber. After 24h, the ESCC cells on the permeable membrane were fixed and stained with crystal violet. The membrane was dried before microscopic evaluation.

### 2.7 Cell proliferation, colony formation, and apoptosis assay

Proliferation: The viability of ESCC cells was determined with a Cell Counting Kit 8 (CCK8, APExBio, Houston, TX, USA). 5 × 10^3^ of ESCC cells were seeded into each well of a 96‐well plate. The change in cell number was determined every 24 hours. Cell density was determined by addition of CCK8 and measuring OD450 nm with a microplate reader. Colony formation: After transfection or drug treatment, 500 ESCC cells were plated in six‐well plates each; after 10 days, the cells were fixed with 4% paraformaldehyde and stained with 1% crystal violet (Sigma‐Aldrich, China). More than 30 cells were considered as a colony. Apoptosis: Tumor cells were pretreated or transfected and collected by trypsinization to wash twice with ice‐cold PBS. Then, then cells were stained using an FITC Annexin/V Apoptosis Detection Kit (BD Pharmingen TM, USA) and quantified by flow cytometry (BD Biosciences, USA).

### 2.8 Luciferase reporter assay

For reporter assays, HEK‐293T or ESCC cells were cotransfected with luciferase reporter, miR‐19b‐3p mimic or inhibitor and 10 ng Renilla luciferase reporter plasmid using Lipofectamine 3000 (Invitrogen, Carlsbad, CA, USA). At 24 h post‐transfection, a luciferase assay kit (Promega, Madison, WI, USA) was performed to measure the luciferase and Renilla activity of the cells based on the manufacturer's instructions.

### 2.9 RNA extraction, RNA‐seq, and quantitative RT‐PCR

Using a RNeasy Mini Kit (Qiagen, Valencia, California), total cellular RNA was extracted from cells. The eukaryotic and prokaryotic mRNA was enriched by Oligo (dT) beads or Ribo‐Zero^TM^ Magnetic Kit (Epicentre, Madison, WI, USA). Then, mRNA was transcripted into cDNA to be synthesized by DNA polymerase I, RNase H, dNTP, and buffer. RNA was purified and sequenced using Illumina Novaseq 6000 by Gene Denovo Biotechnology Co. (Guangzhou, China). Quantitative RT‐PCR was performed according to previous published papers using the SYBR system (Takara, China) [15]. The mRNA levels in cell and tissue lysates were normalized against 18S. The miRNA expression in cell and tissue lysates was normalized against U6. Sequences of primers used for qRT‐PCR in this study are listed in **Table S2**.

### 2.10 Co‐immunoprecipitation (Co‐IP)

Co‐IP was performed using indicated antibodies and protein A/G‐conjugated Dynabeads (Invitrogen) according to the manufacturer's instructions. In brief, cell lysates were incubated with indicated antibodies or IgG overnight at 4°C. Then, the protein A/G‐conjugated beads were added into the lysate at 4°C for 2 h. Then, the beads were washed by PBS or lysis buffer and boiled in SDS loading buffer. Western blotting was used to detect the immunoprecipitated proteins.

### 2.11 Chromatin immunoprecipitation (ChIP) assays

ChIP assays were essentially performed using Merck EZ‐ChIP^TM^ KITS (Merck Millipore, USA) according to the manufacturer's instructions. 1 × 10^7^ cells were fixed with 1% formaldehyde (Sigma) for 10 min at room temperature, then stopped with the 1.25 M glycine and incubated for 5 min. The cells were collected, and the sonication step was performed. ChIP dilution buffer and protein G agarose were added and incubated at 4°C for 1h. Then, the antibodies STAT3 and IgG were added and incubated at 4°C overnight. After elution and purification, ChIP DNA was analyzed by qPCR using the primers specified in Table S2.

### 2.12 Exosome extraction, identification, and labeling

Exosomes from patient plasma or culture supernatant were collected through standard centrifugation steps or ExoQuick Plasma prep and Exosome precipitation kit (SBI, USA) according to the manufacturer’s protocol. The size and concentration of the exosomes were quantified using nanoparticle tracking analysis (NTA) and transmission electron microscopy (TEM). The expression of exosomes biomarkers including TSG101, CD9, and CD63 was detected by western blot. For exosomal RNA extraction, exosomes were pretreated with RNase K and normalized against exogenous λ polyA (Takara, China) for qPCR. For exosomal labeling, PHK67 (Invitrogen, China) was added into exosome suspension at 1 mM and incubated for 20 min, then washed by Exosome Spin Columns (MW3000) (Invitrogen, USA). Transwell membranes (pores 0.4 mm) were used for coculture assay. ESCC cells were visualized by fluorescent microscopy after 24 h.

### 2.13 Animal studies

The ethics and protocol were approved by the animal institute of Sun Yat‐Sen University Cancer Center and Shantou University Medical College. For *in vivo* experiments, indicated treatment and control ESCC cells (1 × 10^7^) mixed with Matrigel were injected into the left or right oxter flank of 5‐week‐old BALB/C nude mice, respectively. Tumor formation was monitored every 7 days for total four weeks. After the mice were sacrificed, the tumor volume and weight were measured. Tumor volume = 0.5 × length×width^2^.

### 2.14 Statistical analysis

Data are expressed as mean ± SD of at least three separate experiments. Statistical analyses were performed with SPSS 22.0 software (SPSS, IBM, USA). Student’s t‐test, Pearson correlation, Kaplan–Meier survival analysis, univariate and multivariate Cox proportional hazards method were performed. A *P*‐value of ≤ 0.05 was considered significant.

## 3 Results

### 3.1 MAP2K3 inhibits cell proliferation and invasion in ESCC both in vitro and in vivo

**Fig. 1:**
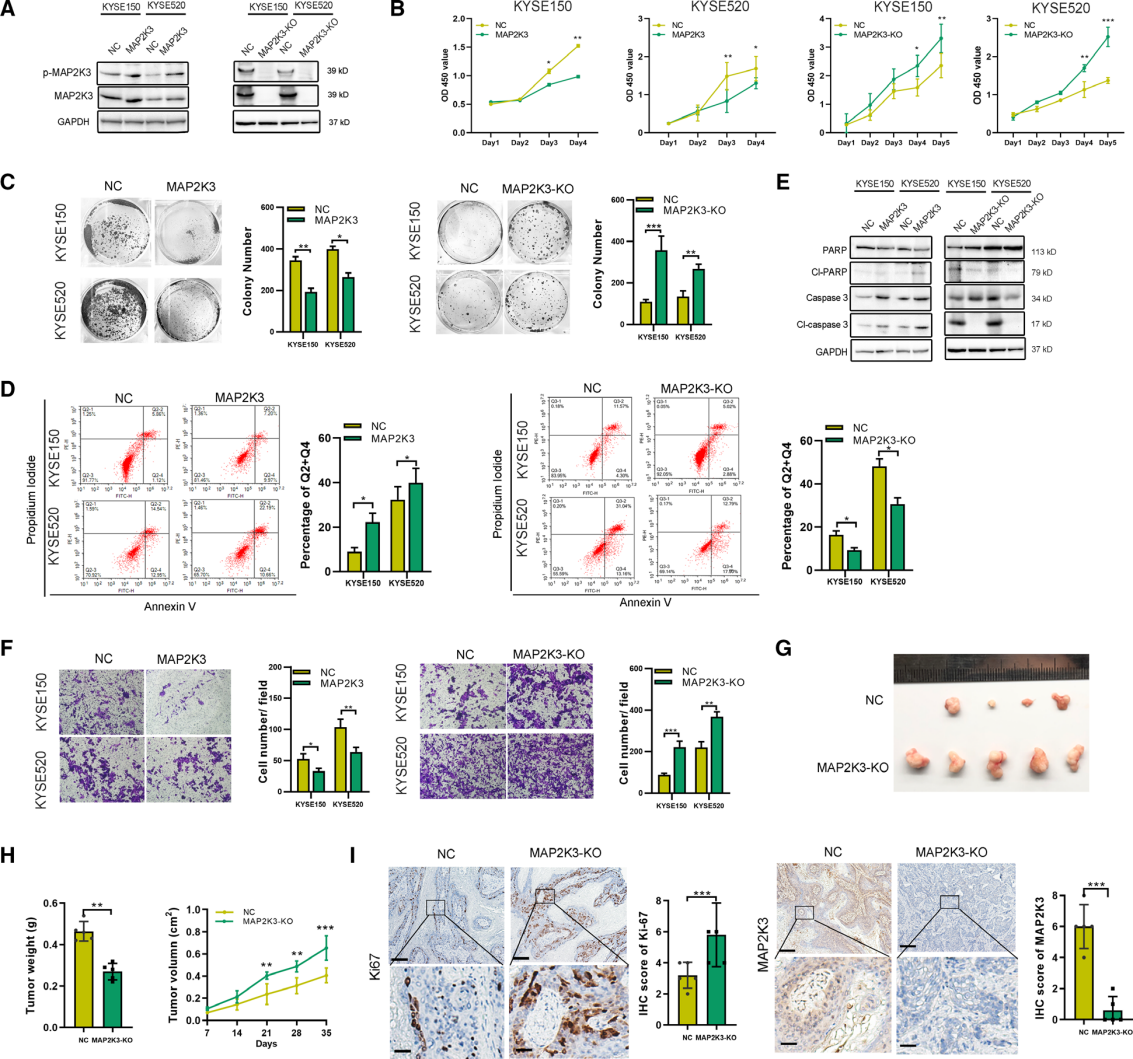
MAP2K3 inhibited cell proliferation and invasion in ESCC in vitro and in vivo. (A) Expression of p‐MAP2K3 and MAP2K3 was detected by western blot in KYSE150 and KYSE520 cells after MAP2K3 transfection or knockout. (B) Cell growth was detected by CCK8 after MAP2K3 transfection or knockout in KYSE150 and KYSE520 cells. (C) Colony formation assay was performed after MAP2K3 transfection or knockout in KYSE150 and KYSE520 cells. (D) Flow cytometry analysis of cell apoptosis caused by MAP2K3 transfection or knockout in KYSE150 and KYSE520 cells. (E) Western blot assay was performed to detect apoptosis biomarkers, cleaved (cl‐) PARP, and caspase 3, after MAP2K3 transfection or knockout in KYSE150 and KYSE520 cells. (F) Cell invasion ability was detected by Transwell assay after MAP2K3 transfection or knockout in KYSE150 and KYSE520 cells. (G) Six weeks after KYSE520 MAP2K3‐KO and control cells were inoculated into the armpits of nude mice (*n* = 5 each group). Tumor volume and mouse weight were measured after injection of the indicated ESCC cells. (H) The tumor weight and size was measured in the indicated time after injection. (I) The representative photographs of immunohistochemistry staining of MAP2K3 and Ki67 in tissues from control or MAP2K3‐KO groups of mice (scale bar: 400 µm, 50 µm, respectively). Error bars represent the SD from at least three independent biological replicates. (*P < 0.05; **P < 0.01; ***P < 0.001 by Student’s *t*‐test)

To identify the kinase responsible for cell survival, we transduced three ESCC cell lines, KYSE520, KYSE150, and TE1, with a CRISPR knockout (GeCKOv.2) library, targeting the kinome, at a multiplicity of infection (MOI) < 0.3. We exposed transduced and control tumor cells for 5 weeks in independent screens and observed that the distribution of the sgRNA reads in library transfected samples compared with that of controls was significantly altered in screens (Figure S1A, B). We selected the candidate genes based on the RNAi Gene Enrichment Ranking metric and identified 5 genes, CRKL, MAP2K3, GK5, CERK, and WEE2, identified as hits by multiple sgRNAs. These selected genes were subjected to loss‐of‐function analysis in ESCC cells by sgRNA. Notably, interference of MAP2K3 suppressed cell proliferation compared with the remaining four genes (Figure S1C). Therefore, we focused on MAP2K3, an important gene in mitogen‐activated protein kinase (MAPK) signaling. To support the functional role of MAP2K3, we measured cell proliferation, colony formation, and invasion ability after MAP2K3 transfection. As shown in Fig. 1A, MAP2K3 transfection increased the expression of Ser‐189 and Thr‐193 phosphorylation (p‐MAP2K3) in ESCC cell lines, KYSE150 and KYSE520. Both ESCC cells with MAP2K3 transfection showed a significant decrease in cell proliferation and colony formation detected by CCK8 and colony formation assay, respectively (Fig. 1B,C). MAP2K3 transfection increased apoptosis compared with the negative control cells, as detected by flow cytometry and western blot (Fig. 1D,E). The cell invasion ability of ESCC cells was decreased after transfecting compared with control (Fig. 1F). Meanwhile, silencing of MAP2K3 resulted in the opposite behavior in ESCC cells (Fig. 1B–F).

The MAP2K3 protein kinase is activated by dual phosphorylation on Ser‐189 and Thr‐193; therefore, we detected whether these phosphorylation sites played a role in the function of MAP2K3 in ESCC. We constructed the dominant‐negative MAP2K3(S‐A) plasmid and constitutively activated MAP2K3(S‐E) by the replacing of Ser‐189 and Thr‐193 with Ala‐189 and Ala‐193 or Glu‐189 and Glu‐193. As shown in Figure S2A, transfection of MAP2K3(S‐A) significantly increased the total MAP2K3 but not p‐MAP2K3 expression, as detected by immunofluorescence. Compared to the control group, the MAP2K3(S‐E) plasmid transfection inhibited cell growth, colony formation, and cell invasion, while increase the cell apoptosis in ESCC (Figure S2B–F). However, MAP2K3(S‐A) abolished the antitumor effect, suggesting that Ser‐189 and Thr‐193 phosphorylation sites affect MAP2K3’s function on cell proliferation and invasion in ESCC.

To determine the effect of MAP2K3 on ESCC function *in vivo*, we established a stable MAP2K3‐knockout (KO) cell line by using CRISPR/Cas9 in KYSE520 cells. Control or KO cells were then injected into the flanks of nude mice (*n* = 5 each group). Tumors in the KO group grew faster and heavier than in the control group (Fig. 1G,H). In addition, IHC was performed to detect the tumor growth in each group by Ki67 and MAP2K3 staining. The expression of Ki‐67 in the MAP2K3‐KO group was significantly higher than in the control group (Fig. 1I). Collectively, these *in vitro* and *in vivo* data demonstrated that loss of MAP2K3 promotes cell proliferation and invasion in ESCC cells.

### 3.2 MAP2K3 is downregulated in ESCC and correlates with patient’s survival

**Fig. 2:**
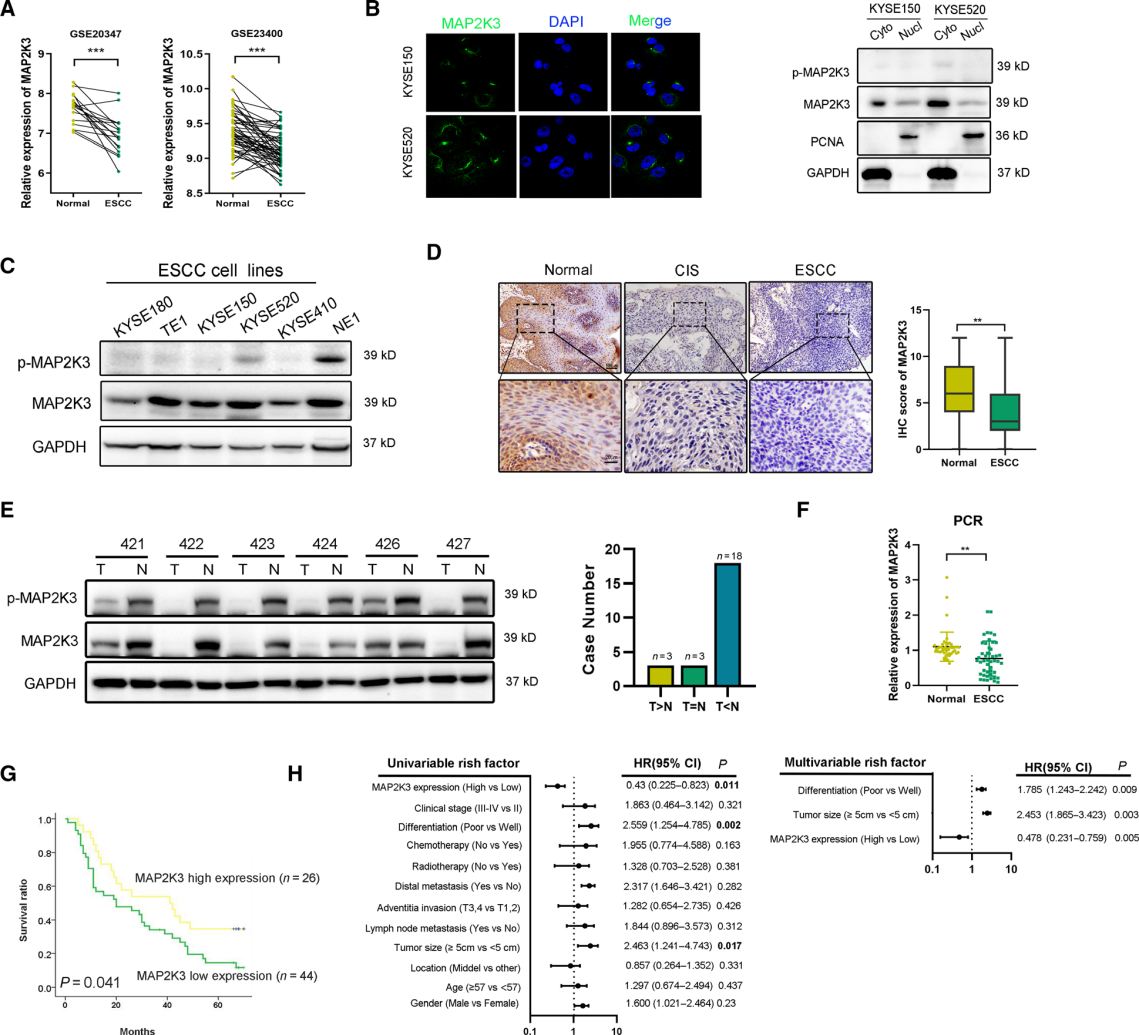
Expression of MAP2K3 in ESCC tissues and its clinical parameters. (A) Expression of MAP2K3 in ESCC and case‐matched normal epithelium was explored in the GSE database (GSE20347 *n* = 34 and GSE23400 *n* = 106). (B) MAP2K3 expression was detected in KYSE150 and KYSE520 by immunofluorescence and nucleus/cytoplasmic assay. (C) Western blot for p‐MAP2K3 and MAP2K3 in ESCC cell lines (KYSE180, TE1, KYSE150, KYSE520, and KYSE410) and an immortalized esophageal cell line (NE1). (D) Representative IHC detection of MAP2K3 in ESCC (*n* = 140), carcinoma in situ (CIS) (*n* = 12), and normal epithelial tissues (*n* = 140). Immunostaining of MAP2K3 in ESCC and normal groups was scored (scale bar: 100 µm, 20 µm, respectively). (E) Western blot for protein expression of MAP2K3 in ESCC (T) and case‐matched normal (N) tissues (*n* = 24). (F) MAP2K3 was detected in 34 pairs of ESCC and case‐matched normal esophageal epithelial tissues by qRT‐PCR. (G) Kaplan–Meier analysis showed that ESCC patients with high levels of MAP2K3 expression (*n* = 26) had longer survival times compared with low MAP2K3‐expressing patients (*n* = 44) (*P* = 0.041, log‐rank test). (H) Forest plot showing the association between MAP2K3 expression and ESCC survival using univariate and multivariate analyses (HR, hazard ratio; CI, confidence interval). Error bars represent the SD from at least three independent biological replicates. (*P < 0.05; **P < 0.01; ***P < 0.001 by Student’s *t*‐test).

To further support the function of MAP2K3 in ESCC, we investigated the expression of MAP2K3 in a cohort of ESCC samples. First, for the GSE20347 (*n* = 34) and the GSE23400 (*n* = 106) cohorts in the GEO database, the expression of MAP2K3 was significantly lower in ESCC than in paired nontumor tissues (Fig. 2A). Next, we found MAP2K3 was primarily expressed in the cytoplasm of ESCC cells, as detected by immunofluorescence. This result was further supported by examining nuclear and cytoplasmic extracts from in ESCC cell lines (Fig. 2B). Then, we identified MAP2K3 expression in six esophageal cell lines, including five ESCC cell lines KYSE150, KYSE520, TE1, KYSE410, and KYSE180, and the NE1 immortalized esophageal epithelial cell line. Compared to NE1, the expression of p‐MAP2K3 and total MAP2K3 was lower in ESCC cells (Fig. 2C). As shown in Fig. 2D, elevated protein expression of MAP2K3 was observed by IHC in normal epithelial (*n* = 140) when compared to case‐matched carcinoma in situ (CIS) (*n* = 12) and ESCC tissue(*n* = 140). By western blot, MAP2K3 protein expression was lower in ESCC compared with the case‐matched normal epithelial tissues in the majority of ESCC cases (18/24) (Fig. 2E). Consistent with the protein expression, the MAP2K3 mRNA expression level was significantly lower in ESCC compared with the normal epithelial cells (Fig. 2F). Furthermore, MAP2K3 expression was significantly correlated with ESCC patient age and tumor differentiation, with well‐differentiated tumors displaying stronger MAP2K3 expression than poorly differentiated tumors (Table 1). Survival data were available in 70 cases, and Kaplan–Meier analysis indicated that ESCC patients with high MAP2K3 expression tended to have a better overall survival, which is consistent with the survival data from TCGA (*P* = 0.11, log‐rank test, Fig. 2G and Fig. S3A, B). Moreover, univariate and multivariate Cox regression analyses further confirmed MAP2K3 expression as an independent predictor of survival in ESCC patients (Fig. 2H).

**Table 1:** The correlation between MAP2K3 expression and clinical pathology parameters in ESCC

Parameters		Case *n* = 140	Expression		Result
			High(*n* = 52)	Low(*n* = 88)	
Age	≤57	62	14	48	
	>58	78	38	40	*P* < 0.01*
Gender	male	106	44	62	
	female	34	8	26	*P* = 0.264
Differentiated	poor	48	28	20	
	mid	86	26	60	
	well	6	6	0	*P* < 0.01*
Location	Upper	16	8	8	
	Middle	108	38	70	
	Lower	16	6	10	*P* = 0.879
Tumor size	>5cm	46	16	30	
	<5cm	94	36	58	*P* = 0.31
Clinical stage	II	74	32	42	
	III	62	20	42	
	IV	4	0	4	*P* = 0.442
Adventitia invasion	T1–T2	32	14	18	
	T3–T4	108	38	70	*P* = 1
Lymph node metastasis	No	78	38	40	
	Yes	62	22	40	*P* = 0.337
Distal metastasis	No	136	52	84	
	Yes	4	0	4	*P* = 0.502

### 3.3 MAP2K3 inhibits the EGFR/STAT3 signaling pathway by promoting STAT3 proteasome degradation

**Fig. 3:**
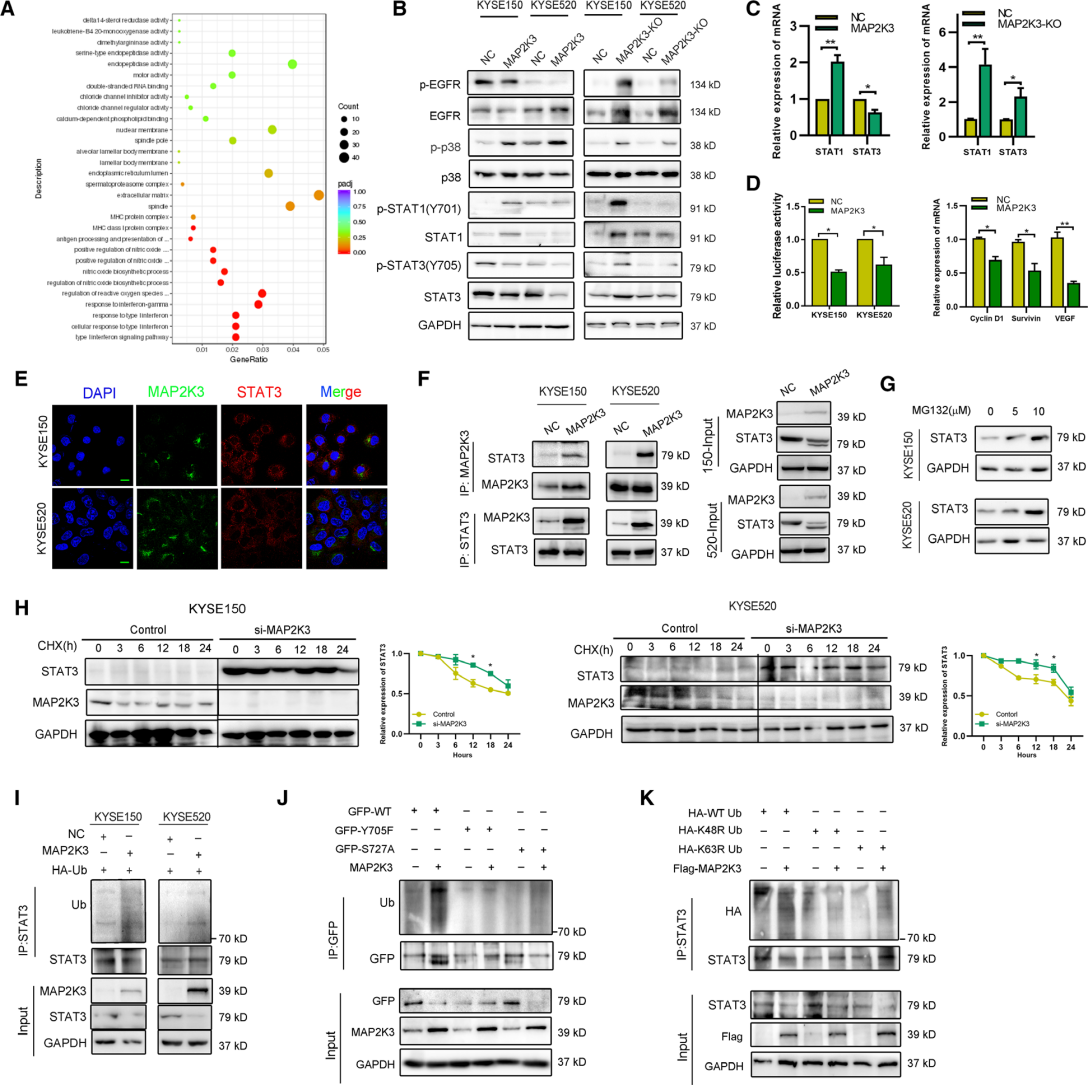
MAP2K3 modulates the EGFR/STAT3 signaling pathway in ESCC by promoting its proteasome degradation. (A) Distribution of the top 20 enriched GO terms in biology process, cellular component, and molecular function for the differentially expressed genes in MAP2K3‐overexpressing ESCC cells based on RNA‐seq analysis. (B) Immunoblot analysis to detect phosphorylation and total EGFR, p38, STAT1, and STAT3 protein expression in ESCC cells. (C) qPCR analysis to detect STAT1 and STAT3 RNA expression after MAP2K3 knockout and transfection in KYSE520 cells. (D) The transcription activity of STAT3 was detected after MAP2K3 transfection by luciferase reporter assay (left panel). The mRNA expression of STAT3 downstream genes was detected by qRT‐PCR (right panel). (E) Colocalization of STAT3 and MAP2K3 was detected by immunofluorescence in KYSE150 and KYSE520 (scale bar: 20 µm). (F) Binding of endogenous MAP2K3 with STAT3 was detected by co‐immunoprecipitation in KYSE150 and KYSE520 cells. (G) Expression of STAT3 was detected by western blot after different doses of MG132 treatment for 24 h. (H) ESCC cells were treated by cycloheximide (CHX, 200 µg·mL^−1^) in a time‐dependent manner after transfecting si‐MAP2K3 and control. **I.** STAT3 ubiquitination was detected after MAP2K3 transfection by immunoprecipitation with anti‐STAT3 antibody and immunoblotting with anti‐Ub. (J) GFP‐STAT3 (WT), GFP‐STAT3 (Y705F), or GFP‐STAT3 (S727A) were transfected into KYSE150 cells together with the MAP2K3 plasmid or control, then STAT3 ubiquitination was detected. (K) HA‐tagged wild‐type, K48R, and K63R Ub were transfected into KYSE150 cells together with the MAP2K3 plasmid. STAT3 ubiquitination was detected. Error bars represent the SD from at least three independent biological replicates. (*P < 0.05; **P < 0.01; ***P < 0.001 by Student’s *t*‐test).

We next explored the mechanisms mediated by MAP2K3 in ESCC. RNA‐seq was performed to identify differentially expressed genes between MAP2K3‐overexpressing and control cells in three ESCC cell lines, KYSE150, KYSE520, and TE1. There were 693 upregulated genes and 304 downregulated genes and 9 of which were identified in all cell lines after applying a twofold screening filter (Figure S4A). Seven of the overlapping differentially expressed genes were subjected to validation by qRT‐PCR (fold change > 3, Figure S4B). Notably, Gene Ontology (GO) analysis revealed that the type I and II interferon (IFN) signaling pathway was one of the most significantly activated pathways mediated by MAP2K3 in ESCC (Fig. 3A). Overexpression of MAP2K3 increased p38 and STAT1, while decreased EGFR and STAT3 expression. However, knockdown of MAP2K3 also increased both p38 expression and STAT1 expression in ESCC cells, indicating that EGFR and STAT3 are regulated by MAP2K3 in ESCC (Fig. 3B,C). Since STAT3 is an important transcription factor in tumorigenesis, we further detected the effect of MAP2K3 on STAT3 transcription activity. As shown in Fig. 3D, MAP2K3 decreased STAT3 transcription activity and the mRNA expression of downstream STAT3 genes, such as cyclin D1, survivin and vascular endothelial growth factor (VEGF).

We subsequently further explored how MAP2K3 mediates expression of STAT3 in ESCC. First, we found that MAP2K3 and STAT3 were mainly colocalized in the cytoplasm as detected by immunofluorescence confocal microscopy (Fig. 3E). Furthermore, bands specific to MAP2K3 or STAT3 were obtained by co‐immunoprecipitation (co‐IP) assay and subjected to western blot analysis for protein identification. STAT3‐MAP2K3 interaction was higher in the MAP2K3‐overexpressing group than in the control group (Fig. 3F). The coiled‐coil domain of STAT3 was crucial for binding to MAP2K3 (Figure S4C). Previous research demonstrated that STAT3 is downregulated in a ubiquitin–proteasome degradation‐dependent manner. Treatment of ESCC cells with MG132 resulted in increased expression of the STAT3 protein (Figure 3G). Then, we detected whether MAP2K3 affects ubiquitination and degradation of the STAT3 protein. The half‐life of STAT3 was significantly increased in MAP2K3 knockdown cells as detected by cycloheximide chase assay (Figure 3H). Ubiquitination assay suggested that STAT3 protein ubiquitination was increased in MAP2K3‐overexpressing cells as compared to the control cells (Figure 3I). Tyrosine 705 (Y705) and serine 727 (S727) phosphorylation sites on STAT3 play an important role in STAT3 degradation. We found that MAP2K3 binds with both wild‐type and mutant STAT3 but did not increase the polyubiquitination of STAT3 S727A (Figure S4D and Figure S3J). MAP2K3 increased ubiquitination of STAT3 on the K48‐linked ubiquitin chain, but not the K63‐linked ubiquitin chain (Figure 3K). These findings suggest that the degradation of STAT3 mediated by MAP2K3 is dependent on the STAT3 Y705 phosphorylation site.

### 3.4 MAP2K3 interacts with the E3 ligase MDM2 to promote STAT3 degradation

**Fig. 4:**
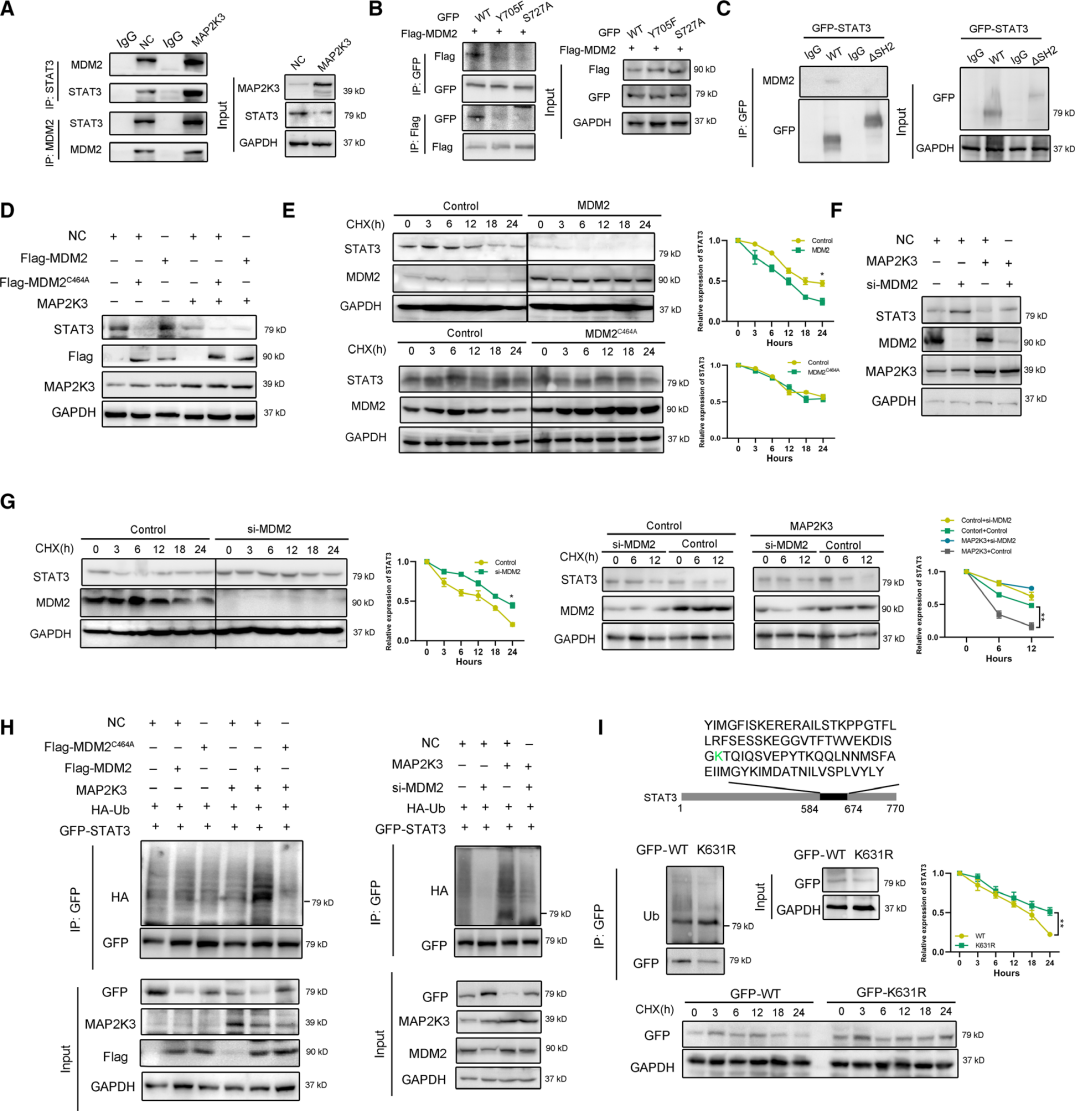
MAP2K3 interacted with E3 ligase MDM2 to promote STAT3 degradation. (A) The interaction of STAT3, MAP2K3, and MDM2 was detected by co‐immunoprecipitation in KYSE150 cells. (B) The interaction of GFP‐tagged STAT3 wild‐type (WT), Y705F, S727A, and Flag‐MDM2 was detected by co‐immunoprecipitation in 293T cells. (C) The interaction of GFP‐tagged STAT3 wild‐type (WT) or SH2 domain deletion (ΔSH2) and MDM2 was detected by co‐immunoprecipitation in KYSE150 cells. (D) MDM2 decreased STAT3 protein. KYSE150 cells were transfected with Flag‐MDM2 or Flag‐MDM2^C464A^ as well as control or MAP2K3 transfection. The protein expression level of STAT3 was assayed by western blot. (E) The cells expressing MDM2 or MDM2^C464A^ were treated with cycloheximide (CHX, 200 µg·mL^−1^). The protein levels of STAT3 and MDM5 were analyzed by western blot. (F) Knockdown MDM2 increased STAT3 protein. KYSE150 cells were transfected with si‐MDM2 as well as control or MAP2K3 transfection. (G) KYSE150 cells were transfected with control or MDM2 siRNAs treated with CHX, and the protein levels of STAT3 and MDM2 were analyzed by western blot. (H) MDM2 ubiquitylates STAT3. KYSE150 cells were transfected with indicated plasmids or siRNA for 48 h. (I) KYSE150 cells were transfected with indicated plasmid, and western blot was performed to analyze the expression of indicated proteins and ubiquitination. Error bars represent the SD from at least three independent biological replicates. (*P < 0.05; **P < 0.01; ***P < 0.001 by Student’s *t*‐test).

We next investigated which E3 ubiquitin ligase mediates STAT3 protein degradation induced by MAP2K3. Mouse double minute 2 (MDM2), which located in both cytoplasm and nucleus of ESCC cells, was predicted as the highest confidence primary E3 ligase for STAT3 in the UbiBrowser database (Figure S5). We then confirmed a stronger interaction between STAT3 and MDM2 after MAP2K3 transfection (Figure 4A), suggesting that MDM2 associates with STAT3 in ESCC cells. Moreover, the tyrosine 705 (Y705) and serine 727 (S727) phosphorylation site mutations of STAT3 abolished the observed interactions with MDM2 (Figure 4B). The SH2 domain deletion of STAT3 also abolished the interactions (Figure 4C). Taken together, our data suggested that MDM2 binds to STAT3 SH2 domain.

Then, we found that STAT3 expression was sharply reduced after overexpressed wild‐type (wt) MDM2 in KYSE150 cells. However, ectopic expression of MDM2^C464A^, which lacks ubiquitin ligase activity, did not affect levels of STAT3 (Figure 4D). Consistently, the half‐life of STAT3 was significantly reduced in MDM2 overexpressing cells but not in MDM2^C464A^‐overexpressing cells (Figure 4E). These results suggest that MDM2 is the E3 ligase that mediated STAT3 protein stability in ESCC cells. To investigate whether MDM2 contributes to MAP2K3‐induced STAT3 protein degradation, we knockdown MDM2 in KYSE150 and found that si‐MDM2 increases STAT3, and this effect of MDM2 was more substantial after MAP2K3 transfection (Figure 4F). Consistently, knockdown of MDM2 extended the half‐life of STAT3, and the effect of MDM2 was more significant after MAP2K3 transfection (Figure 4G). The ubiquitination assays showed that wild‐type MDM2, not MDM2^C464A^, significantly increased the ubiquitination of STAT3. Consistently, knockdown of MDM2 reduced STAT3 ubiquitination in KYSE150 cells (Figure 4H). By analyzing the lysine residues within the SH2 domain for possible ubiquitination sites, we mutated a predicted K631 lysine residues to Ala and found that STAT3 ubiquitination was abolished (Figure 4I). Taken together, these results demonstrate that MAP2K3 interacts with the E3 ligase MDM2 to promote STAT3 degradation.

### 3.5 STAT3 is an essential factor in MAP2K3‐mediated tumorigenesis in ESCC

**Fig. 5:**
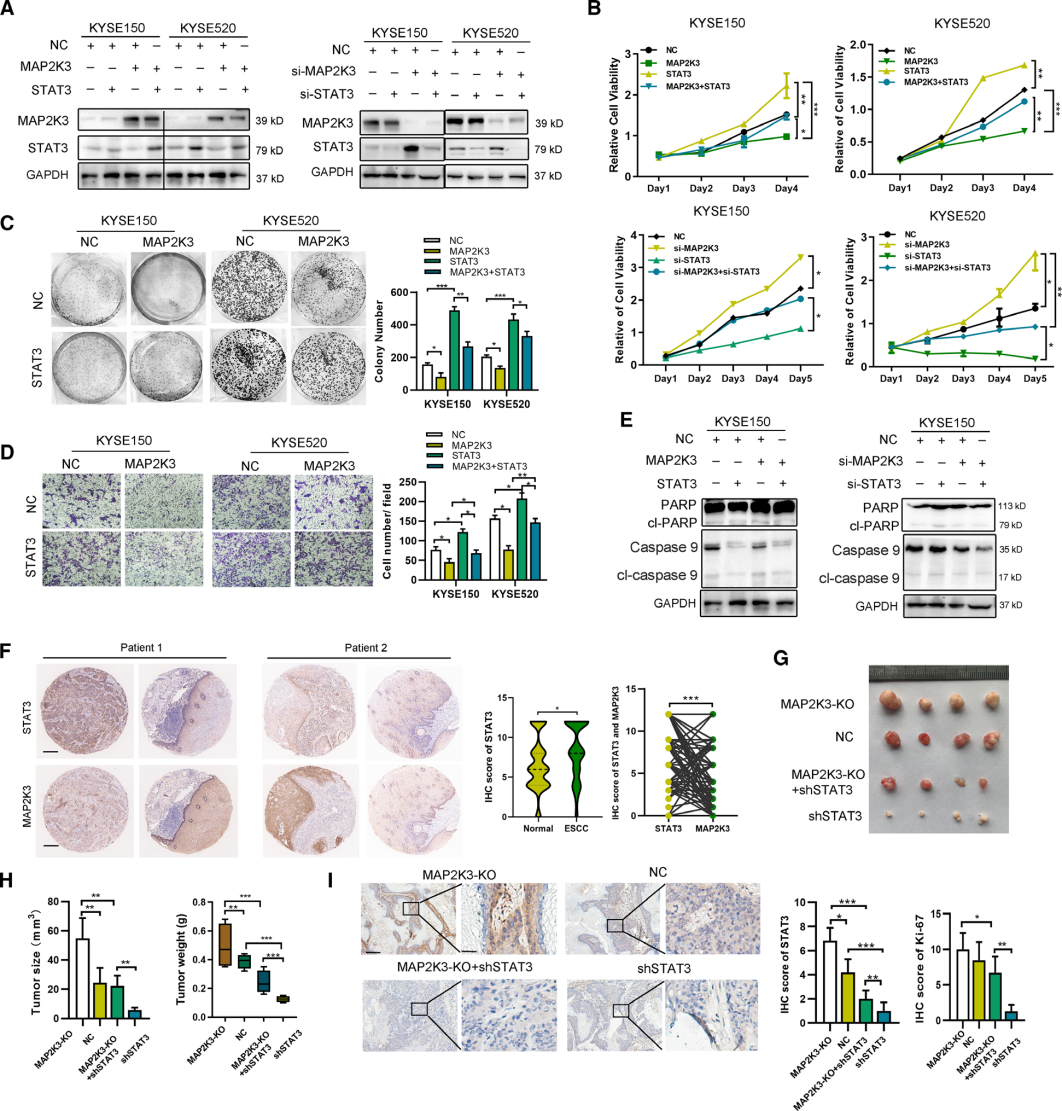
STAT3 is an essential factor in MAP2K3‐mediated tumorigenesis in ESCC. (A) The transfection efficiency of indicated plasmids was detected by western blot. (B) Cell growth was detected by CCK8 in KYSE150 and KYSE520 cells with indicated transfection. (C) Colony formation was detected in STAT3 and MAP2K3 transfected KYSE150 and KYSE520 cells. (D) Cell invasion was detected in STAT3 and MAP2K3 transfection cells by Transwell assay. (E) Western blot was performed to detect the expression of (cl‐) PARP and caspase 9 in KYSE150 cells after indicated transfection. (F) MAP2K3 and STAT3 expression was evaluated by IHC (*n* = 140, scale bars: 200 µm). The IHC score and the correlation between STAT3 and MAP2K3 expression in ESCC patients were shown. (G). The 5‐week‐old immunodeficient nude mice (4 mice per group) were injected subcutaneously with indicated cells (1 × 10^7^ cells). (H). Tumor volume and weight were measured at day 30. (I) Immunohistochemistry analysis of STAT3 and Ki67 of tumor xenografts with indicated treatment (scale bar: 200µm, 50 µm, respectively). Error bars represent the SD from at least three independent biological replicates (*P < 0.05; **P < 0.01; ***P < 0.001 by Student’s *t*‐test).

To investigate whether STAT3 is involved in MAP2K3‐mediated tumorigenesis in ESCC, we transfected ESCC cells with a plasmid encoding STAT3 and treated the cells. Compared to the control cells, STAT3 overexpression significantly promoted KYSE150 and KYSE520 cell proliferation and colony formation (Figure S6A, B), and western blot analysis indicated that STAT3 overexpression significantly diminished the expression of apoptotic biomarkers (Figure S6C). Conversely, STAT3 knockdown significantly attenuated KYSE150 and KYSE520 cell proliferation and clonogenicity. Collectively, these results suggest that STAT3 functions as oncogene in ESCC. We also aimed to detect whether MAP2K3 mediates ESCC cell proliferation and invasion through suppressing STAT3 by performing rescue experiments involving overexpressing STAT3 in MAP2K3‐transfected cells and knockdown of STAT3 in si‐MAP2K3‐transfected cells. Transfection efficiency is shown in Figure 5A. As shown in Figure 5B‐D and Figure S6D, colony formation, cell invasion, and proliferation experiments suggested that overexpression of STAT3 abolished MAP2K3‐inhibited cell proliferation and invasion. Conversely, knockdown of STAT3 by siRNA partially reversed the cell proliferation and invasion ability induced by loss of MAP2K3 (Figure 5E).

To determine whether a clinical correlation exists between MAP2K3 and STAT3 expression, we analyzed their expression in 140 ESCC tissues by IHC staining (Figure 5F). STAT3 expression was 1.53‐fold higher in ESCC compared with case‐matched normal epithelial tissues and was negatively correlated with MAP2K3 expression. Furthermore, the *in vivo* experiment supporting our in vitro results showed that tumor size and weight were significantly higher in MAP2K3‐KO tumors compared with controls, while STAT3 knockdown tumors had the smallest tumors (Figure 5G, H). The immunohistochemistry revealed that STAT3 and Ki‐67 expression was significantly higher in the MAP2K3‐KO group, while knockdown of STAT3 abolished this phenomenon, indicating that STAT3 knockdown diminished tumor activity induced by MAP2K3‐KO (Figure 5I and Figure S6E). Base on the above results, we concluded that STAT3 is an essential factor in MAP2K3‐mediated tumorigenesis in ESCC.

### 3.6 Exosomal miR‐19b‐3p suppresses MAP2K3 to promote ESCC tumorigenesis

**Fig. 6:**
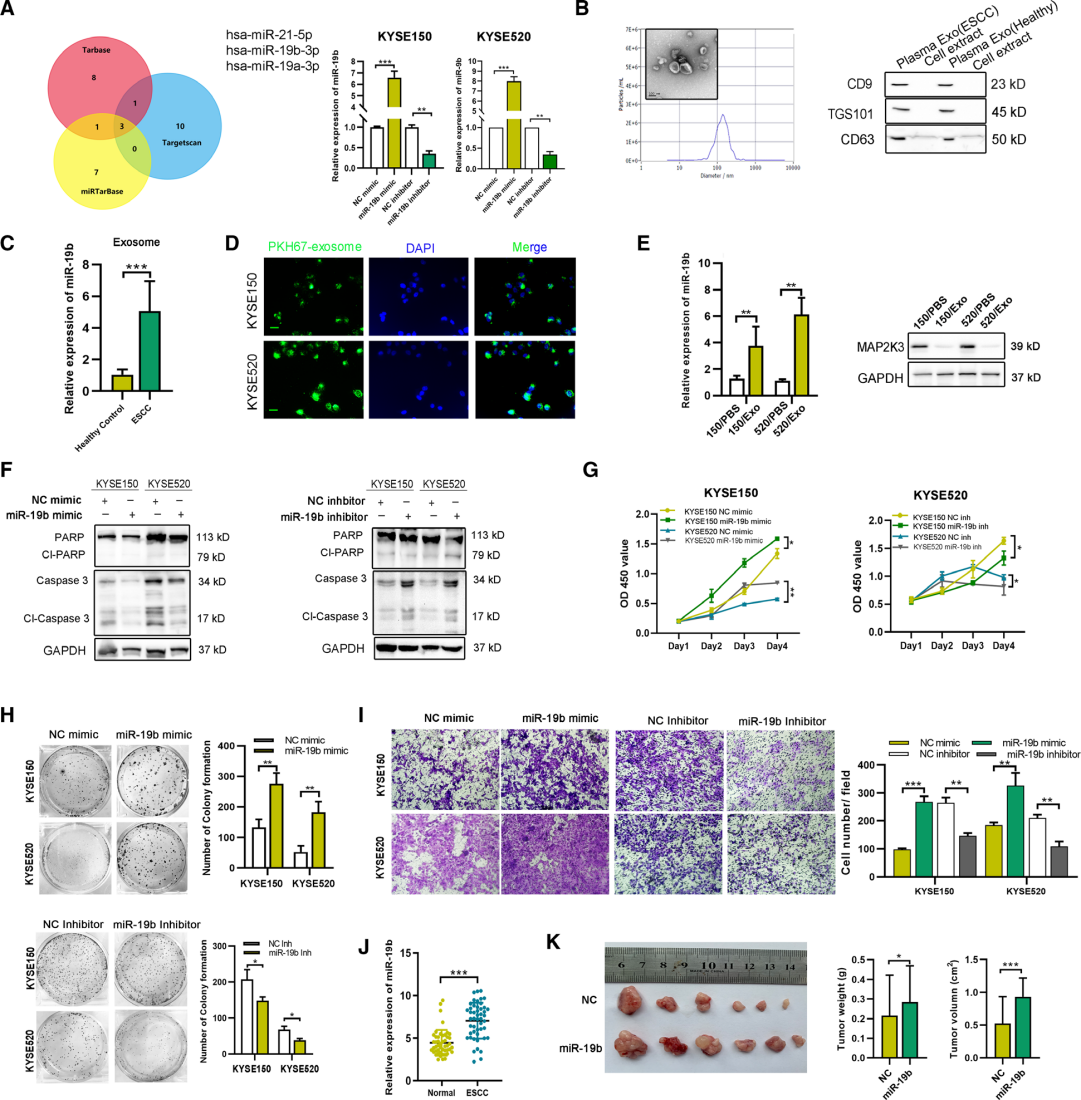
Exosomal miR‐19b‐3p‐mediated cell proliferation and invasion via suppressing MAP2K3. (A) Venn Diagram: Number of predicted miRNAs from TargetScan, DIANA‐TarBase, and miRTarBase is shown, identifying three miRNAs: miR‐21‐5p, miR‐19a‐3p, and miR‐19b‐3p. The transfection efficiency was detected after miR‐19b‐3p mimic or inhibitor transfection. (B) The exosomes were identified using transmission electron microscopy, nanoparticle tracking analysis (NTA), and western blot analysis (scale bar, 100 nm). (C) Exosomal miR‐19b‐3p expression in healthy (*n* = 7) and ESCC patient (*n* = 7) plasma detected by qRT‐PCR. (D) ESCC patient plasma‐derived exosome was dyed with PKH67 (green) and cocultured with ESCC cells for 12 h (scale bar, 100 nm). (E) The expression of miR‐19b‐3p and MAP2K3 in ESCC cells with indicated treatment was detected by qRT‐PCR and western blot. (F) Western blot for apoptosis biomarkers, PARP, and caspase 3 after miR‐19b‐3p mimic transfection. (G) CCK8 assay was performed to detect the cell proliferation in KYSE150 and KYSE520 cells transfected with miR‐19b‐3p mimic, inhibitor, or control vector. (H) Colony formation of KYSE150 and KYSE520 cells transfected with miR‐19b‐3p mimic, inhibitor, or control vector was detected. (I) Cell invasion ability was detected by Transwell assay in KYSE150 and KYSE520 cells transfected with miR‐19b‐3p mimic, inhibitor, or control vector. (J) The expression of miR‐19b‐3p was detected by IHC in ESCC tissues and case‐matched normal esophageal epithelial (*n* = 48). (K) The 5‐week‐old BALB/C nude mice (6 per group) were injected with stable miR‐19b‐3p expression or control cells, and then, the tumor volume and weight were measured after 4 weeks. Error bars represent the SD from at least three independent biological replicates. (*P < 0.05; **P < 0.01; ***P < 0.001 by Student’s *t*‐test).

To uncover the mechanism by which MAP2K3 is downregulated in ESCC, we performed several experiments. However, we found that low expression of MAP2K3 in ESCC is caused by neither proteasome degradation nor promoter methylation (Figure S7A). miRNAs are involved at the posttranscriptional level and bind to the 3' UTRs of their target mRNAs to suppress gene expression. Thus, using predictions of TargetScan, DIANA‐TarBase and miRTarBase databases, we identified 3 miRNAs (miR‐21‐5p, miR‐19a‐3p, and miR‐19b‐3p) in common that could serve as potential upstream regulators of MAP2K3 (Figure 6A). PCR analysis revealed that all three miRNAs mimics decreased MAP2K3 mRNA expression, with miR‐19b‐3p being the most significant (Figure S7B). Using 3′ UTR luciferase reporter assays, we found that a miR‐19b‐3p mimic inhibited luciferase activity of a MAP2K3‐dependent reporter gene, while mutant vectors had no influence, and a miR‐19b‐3p inhibitor induced the opposite effect (Figure S7C). A miR‐19b‐3p mimic suppressed MAP2K3 RNA and protein expression, while a miR‐19b‐3p inhibitor increased MAP2K3 expression (Figure 6A and Figure S7D). These results indicate that miR‐19‐3p represses expression of MAP2K3 through binding its 3’UTR.

Based on the GSE71043 database, exosomal miR‐19b‐3p expression was 4.19‐fold higher in ESCC patient plasma than the healthy controls (Figure S7E). Thus, we hypothesized that ESCC patient’s plasma‐derived exosomal miR‐19b‐3p could transfer into ESCC cells to suppress MAP2K3 expression. We extracted exosome from ESCC patient plasma and identified exosome by western blot, transmission electron microscopy (TEM), and NTA (Figure 6B). Consistent with previous report that the expression of exosomal miR‐19b‐3p was significantly higher in ESCC patient’s plasma than healthy control (Figure 6C). The fluorescence microscope imaging showed that PKH67‐labeled plasma‐derived exosomes could be delivered to ESCC cells (Figure 6D). The expression of miR‐19b‐3p was higher in ESCC cells cocultured with these exosomes than control. Consistently, the expression of MAP2K3 was decreased (Figure 6E). Therefore, the data showed that exosomes from ESCC patient plasma could transfer miR‐19b‐3p into tumor cells. Then, we determined whether miR‐19b‐3p plays a role in ESCC tumorigenesis. First, we transfected the miR‐19‐3p mimic into ESCC cells that exhibit decreased apoptosis and increased cell survival, colony formation, and cell invasion, compared to control cells. ESCC cells transfected with the miR‐19b‐3p inhibitor exhibited the opposite results (Figure 6F‐I), indicating that miR‐19‐3p promotes ESCC tumorigenesis. The rescue experiments result also supported that miR‐19b‐3p promotes ESCC tumorigenesis by suppressing MAP2K3 (Figure S7F). In our cohort ESCC samples, the expression of miR‐19b‐3p was higher in ESCC compared with normal esophageal epithelium and inversely correlation with MAP2K3 as detected by qRT‐PCR (Figure 6J and Figure S7G). The *in vivo* study also supported our *in vitro* findings that tumors in the stable miR‐19b‐3p high expression group grew faster and heavier than in the control group (Figure 6K). Furthermore, the exosome tracing and functional analysis results demonstrated that exosomes extracted from KYSE150 and KYSE520 cells transfected with miR‐19b‐3p mimics can transfer into recipient ESCC cells and significantly increase the proliferation, colony formation ability of recipient ESCC cells (Figure S8). All these results indicated that plasma‐derived exosomal miR‐19b‐3p can promote proliferation and invasion in ESCC cells by suppressing MAP2K3.

### 3.7 STAT3 binds to the MIR19B promoter to increase miR‐19‐3p expression

**Fig. 7:**
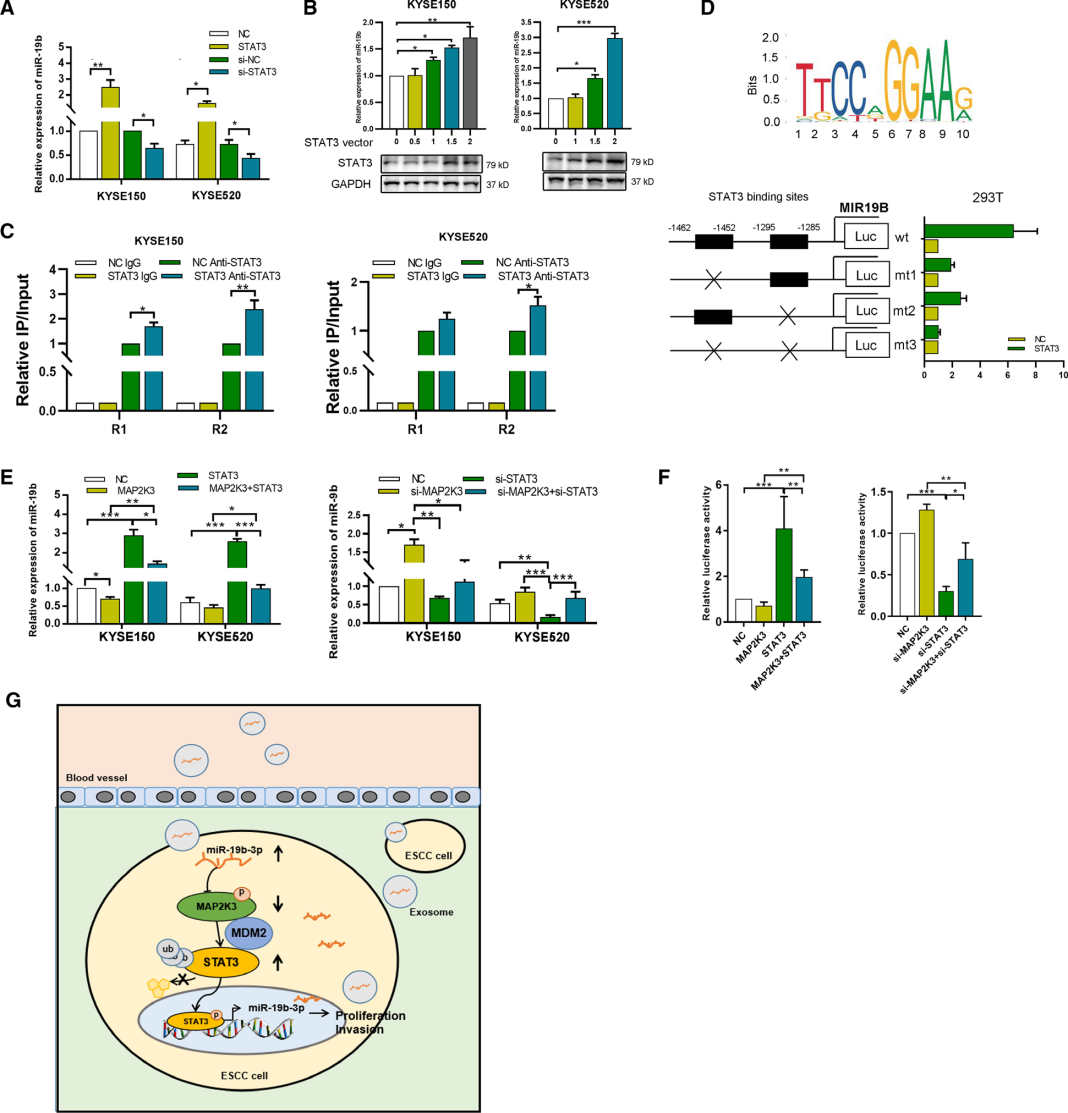
STAT3 binds with miR‐19‐3p promoter to increase miR‐19‐3p expression. (A) The expression of miR‐19b‐3p was detected in STAT3 overexpressed and knockdown KYSE150 and KYSE520 cells. (B) The expression of miR‐19b‐3p was detected in different doses of STAT3 plasmids transfected KYSE150 and KYSE520 cells. (C) ChIP assay was performed to detect the binding of STAT3 in the promoter of MIR19 after STAT3 transfection. (D) The binding motif of STAT3 (upper panel). The HEK‐293T cells were cotransfected STAT3 or control with different combinations of wild‐type (wt) and mutated reporter constructs (mt1, mt2, or mt3). The relative luciferase activity was measured (lower panel). (E) The expression of miR‐19b‐3p was detected in KYSE150 and KYSE520 cells with indicated transfection. (F) The relative luciferase activity of MIR19B was analyzed in the indicated transfected KYSE150 and KYSE520 cells. (G) Schematic model of the role of miR‐19b‐3p/MAP2K3/STAT3 feedback loop in regulating ESCC tumorigenesis. Error bars represent the SD from at least three independent biological replicates. (*P < 0.05; **P < 0.01; ***P < 0.001 by Student’s *t*‐test).

STAT3 is an important transcription factor in tumor progression. Inspection of the MIR19B genomic region revealed a conserved STAT3‐binding according to the JASPAR website (http://jaspar.genereg.net/analysis), and two STAT3 binding sites were identified (‐1462/‐1452 and ‐1295/‐1285 sites). Thus, we examined whether STAT3 directly binds to the MIR19B promoter to increase its expression. First, we analyzed the expression of miR‐19b‐3p expression in response to STAT3 transfection or knockdown. As shown in Figure 7A, STAT3 increased, while si‐STAT3 decreased expression of miR‐19b‐3p in both KYSE150 and KYSE520 cells. We further found that STAT3 overexpression increased expressions of miR‐19b‐3p in a dose‐dependent manner (Figure 7B). To determine whether STAT3 binds to the MIR19B promoter region, specific primers were designed and synthesized for two potential binding sites in MIR19B, and the binding of STAT3 to these putative sites was demonstrated by ChIP analysis. After transfection of KYSE150 and KYSE520 cells with STAT3, the STAT3 occupancy at the MIR19B promoter significantly increased (Figure 7C). To determine whether two STAT3‐binding sites contribute to transcription activity, we constructed four reporter plasmids, wild‐type (WT), mutant (mt)1, mt2, and mt3, with ‐1462‐STAT3 site, ‐1295‐STAT3 site, or both sites mutated, respectively (Figure 7D). These reporter plasmids were cotransfected with STAT3 or control into HEK‐293T cells, and relative luciferase activity was assessed. Compared to the wild‐type, both the ‐1462‐site mutation (mt1) and the ‐1295‐site mutation (mt2) reduced the synergistic activity of STAT3 on promoter activation. Moreover, the synergistic activity of STAT3 was decreased far more significantly in the double‐mutation (mt 3) group (Figure 7D). To further examine MAP2K3/STAT3’s effect on miR‐19b‐3p, we performed rescue experiments and found that expression and transcription activity of miR‐19b‐3p was increased in STAT3 overexpressed cells, while MAP2K3 reduced it. In contrast, STAT3 knockdown decreased expression and transcriptional activity of miR‐19b‐3p, and overexpression of MAP2K3 restored it (Figure 7E, F).

## 4 Discussion

In this study, we show that the miR‐19b‐3p/MAP2K3/STAT3 feedback loop regulates ESCC tumorigenesis. MAP2K3 has lower expression in ESCC than in case‐matched normal esophageal epithelium. MAP2K3 promotes apoptosis and inhibits cell growth in ESCC cells and functionally exerts its effects through the downregulation of the EGFR/STAT3 pathway by promoting STAT3 proteasome degradation. In addition, exosomal miR‐19b‐3p from ESCC patient plasma could be transferred to the ESCC cells and suppress MAP2K3 expression to mediate cell proliferation and invasion. Moreover, STAT3 binds to the MIR19 promoter region and increases expression of miR‐19b‐3p in ESCC cells (Figure 7G).

MAP2K3 is a key mediator of the MAPK signaling pathways and is involved in several biological processes. The role of MAP2K3 in cancer progression is contradictory. MAP2K3 has been shown to enhance tumor progression, and the loss of MAP2K3 results in inhibition of cellular proliferation and increased response of tumor cells to chemotherapeutic drugs *in vivo* [5, 16, 17]. However, recent research has demonstrated that the loss of MAP2K3 copy number occurs in NSCLC and that MAP2K3 inhibits cell proliferation and promotes cellular senescence in hepatocellular carcinoma, breast cancer, and melanoma [7, 18, 19, 20, 21]. In this study, we demonstrated that MAP2K3 is downregulated in ESCC compared with case‐matched normal tissues. We also revealed that ESCC patients with higher MAP2K3 expression have better prognosis. Previous findings have suggested an important role for MAP2K3 in tumor invasion and progression, such as in colon cancer. However, in our study, we found that MAP2K3 inhibits cell proliferation and colony formation, indicating that MAP2K3 plays a tumor suppressor role in ESCC. Loss of MAP2K3 enhances cell proliferation and invasion ability in ESCC.

Furthermore, we demonstrated that MAP2K3 suppresses STAT3 expression in ESCC. STAT3 has been a protein target due to its roles in the progression of cancer development, stemness, chemoresistance, and radioresistance [9]. STAT3 is constitutively activated in cancers, such as ESCC, breast cancer, and lung cancer, which is associated with a poor clinical prognosis [22], but the mechanism has not been clearly demonstrated. In this study, we explored the mechanism underlying MAP2K3‐mediated downregulation of STAT3, performing the protein stability and ubiquitination assays to show that MAP2K3 may directly bind to STAT3 and promote its proteasomal degradation. Furthermore, we revealed that the E3 ligase MDM2 mediates in this process. MDM2 is known to induce ubiquitination of several target proteins, such as p53 [23]. MDM2 upregulation is associated with cancer development by repressing p53 [24]. In this paper, we found that MDM2 interacted with STAT3 to promote STAT3 ubiquitination and this progress was enhanced by MAP2K3 transfection.

In the end, we attempted to identify the mechanisms underlying the downregulation of MAP2K3 in ESCC. miRNAs regulate gene expression by binding the 3’UTR of target transcripts in a complete or incomplete complementary binding manner. According to our predicted results, MAP2K3 might be a target gene of miR‐19b‐3p, a key oncogenic component of the polycistronic miR‐17∼92 cluster [25]. Then, we identified miR‐19b‐3p as an upstream regulator of MAP2K3 by luciferase reporter assay. In cancer, miR‐19b‐3p promotes cell proliferation, invasion, and metastasis in clear cell renal cell carcinoma, lung, colorectal, and breast cancer [26, 27]. High expression of miR‐19b‐3p was found in colon cancer and associated with poor patient survival [28]. However, the role of miR‐19b‐3p is not without controversy. The miR‐19b expression level is significantly downregulated and function as a tumor suppressor in breast cancer [29]. However, the expression and function of miR‐19b‐3p in ESCC development remains unclear. In this study, we reported that miR‐19b‐3p can promote cell proliferation and suppress apoptosis in ESCC by targeting MAP2K3. And the rescue experiments convince us that the function of miR‐19b‐3p is partly dependent on suppressing MAP2K3. Furthermore, we identified STAT3 as the transcription driver of miR‐19b‐3p. We firstly validated that STAT3 upregulates the expression of miR‐19b‐3p and binds with the promoter region of MIR19B to increase its transcription. Thus, miR‐19b‐3p, MAP2K3, and STAT3 formed a positively feedback loop to contribute to ESCC progression.

## 5 Conclusion

In summary, we showed that MAP2K3 was downregulated in ESCC and the low MAP2K3 expression was correlated with clinically poor survival. MAP2K3 inhibited cell proliferation and invasion via the EGFR/STAT3 signaling pathway in ESCC cells. MAP2K3 suppressed STAT3 expression and activation by promoting STAT3 ubiquitin–proteasome degradation. Furthermore, we found that miR‐19b‐3p suppressed MAP2K3 expression and could be transcriptional activated by STAT3 in ESCC cells. Our study is the first to demonstrate that miR‐19b‐3p/MAP2K3/STAT3 feedback loop regulates ESCC tumorigenesis. Therefore, MAP2K3 may serve as a predictor and potential therapeutic target for ESCC.

## Conflict of interests

The authors declare no conflicts of interest.

## Authors contributions

All the authors have precipitated in the conception and design of the study. YZ, WQL, YLC, YBL XY, and ZYL have obtained and analyzed the data. YZ, WQL, YLC, and HW organized the data and drafted the manuscript. YZ and ZYL revised the manuscript. All the authors have read and approved the final version of the manuscript.

## Ethics approval and consent to participate

All authors approved and directly participated in the planning, execution, and/or analysis of the data presented here. All protocols were approved by the Ethics Committee of Shantou University Medical College, and informed consent was obtained from all patients before surgery. All in vivo protocols were approved by the Institutional Animal Care and Use Committee of Shantou University Medical College.

## Consent for publication

The content of this manuscript has not been previously published and is not under consideration for publication elsewhere.

## Supporting information


**Fig S1.** Differentially expressed genes in the CRISPR/Cas9 screen.

**Fig S2.** The function of MAP2K3 in ESCC dependent on its phosphorylation sites.

**Fig S3.** Kaplan‐Meier analysis of MAP2K3 in ESCC patients.

**Fig S4.** The rescue experiments of MAP2K3 and STAT3 in ESCC.

**Fig S5.** Identification of MDM2 as E3 ligase of STAT3 by bioinformatics.

**Fig S6.** The function of STAT3 in ESCC.

**Fig S7.** MAP2K3 was suppressed by miR‐19b‐3p in ESCC cells.

**Fig S8.** Exosomal miR‐19b‐3p transferred in ESCC cells.

**Table S1.** CRISPR/CAS9 kinome gene list.

**Table S2.** Primers.

## Data Availability

The datasets used for the current study are available from the corresponding author on reasonable request. The authenticity of this article has been validated by uploading the key raw data onto the Research Data Deposit public platform (http://www.researchdata.org.cn/), with the approval RDD number as RDDB2020000866.
