# Supplementary figures and images for: The miR‐19b‐3p‐MAP2K3‐STAT3 feedback loop regulates cell proliferation and invasion in esophageal squamous cell carcinoma
Source: Mol Oncol. 2021 Mar 14;15(5):1566–83. doi: 10.1002/1878-0261.12934 (PMC8096789; doi:10.1002/1878-0261.12934)

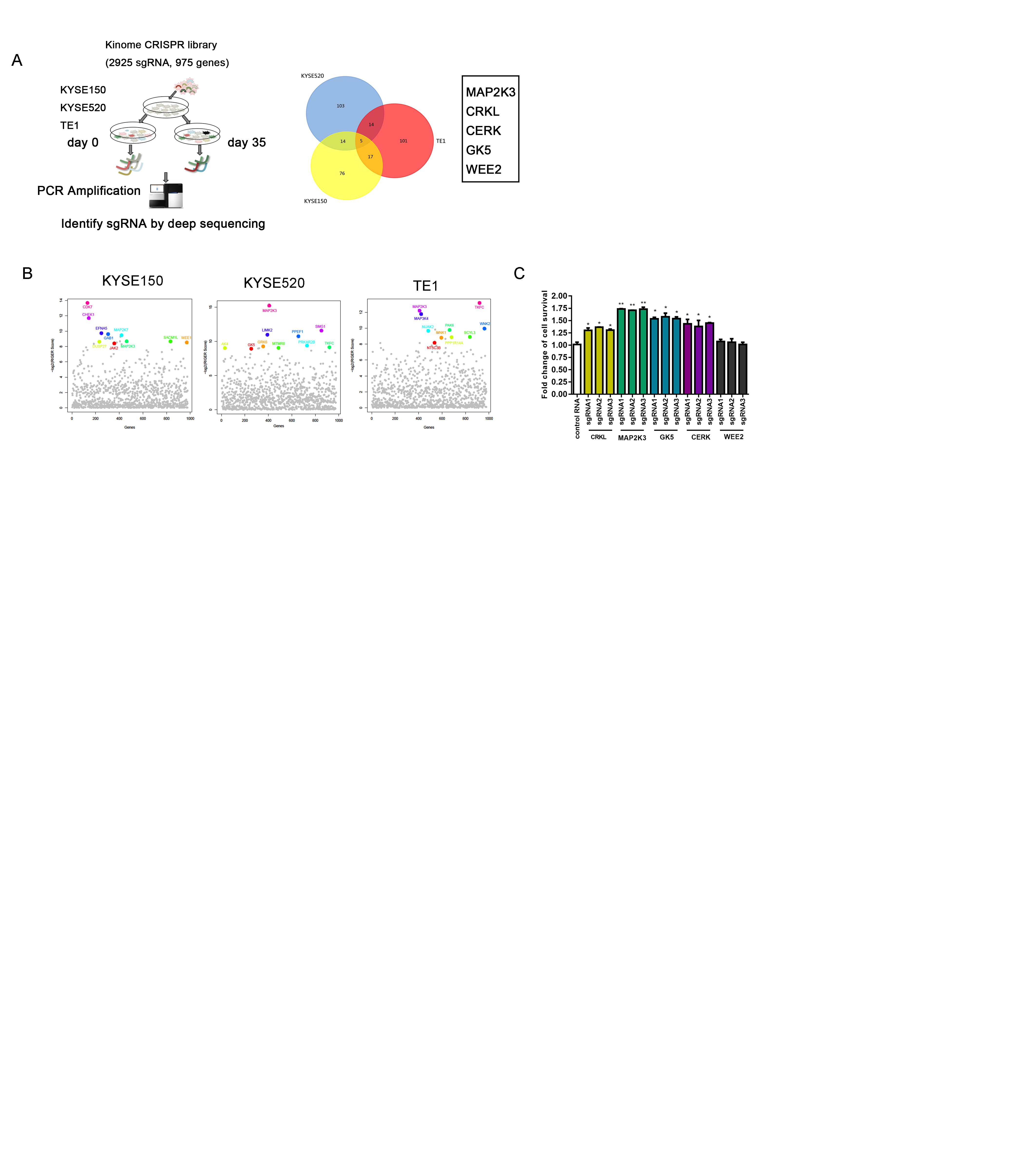

Supplement: Supplementary file 1 — Fig S1. Differentially expressed genes in the CRISPR/Cas9 screen. [file MOL2-15-1566-s005.tif]

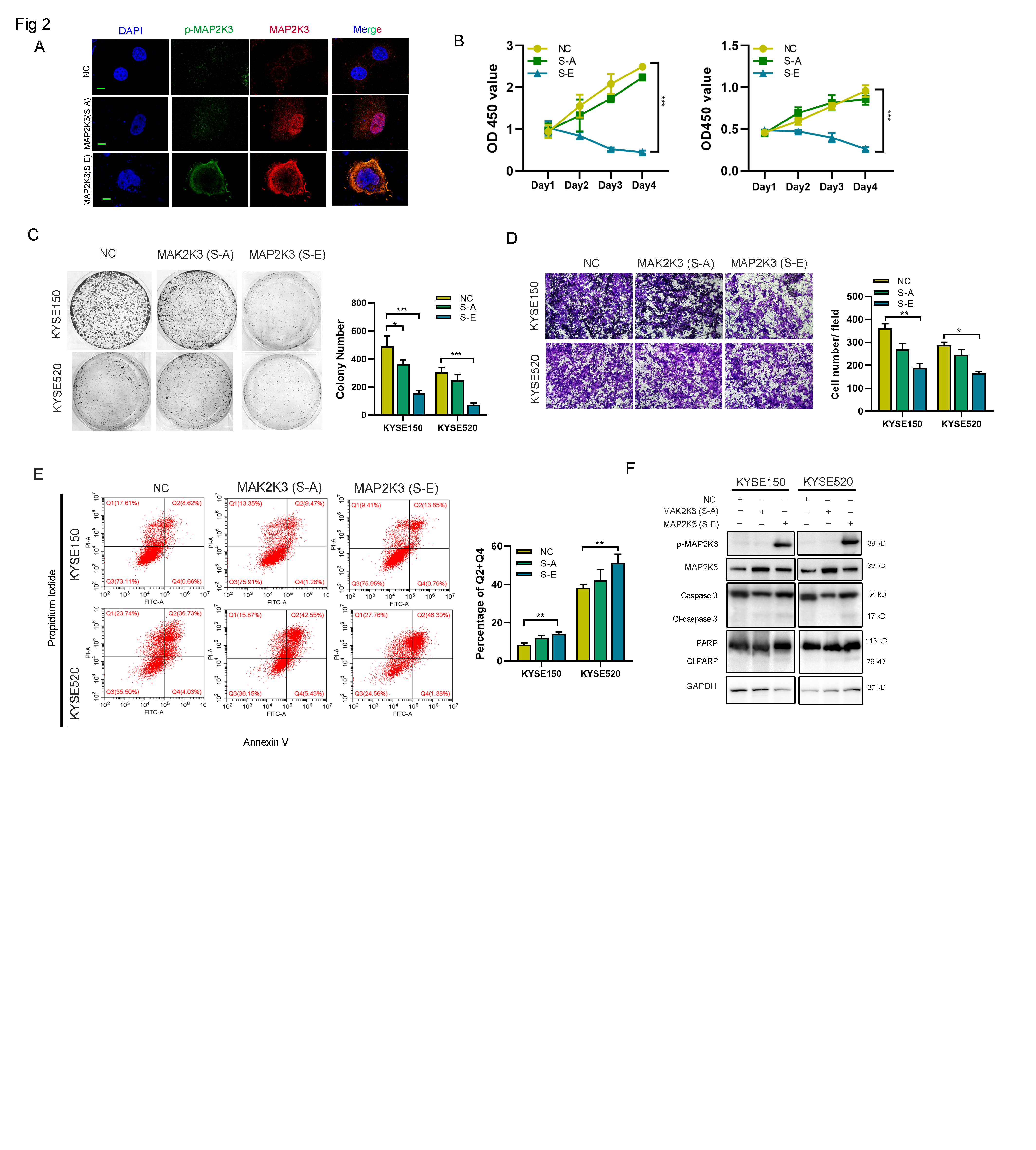

Supplement: Supplementary file 2 — Fig S2. The function of MAP2K3 in ESCC dependent on its phosphorylation sites. [file MOL2-15-1566-s003.tif]

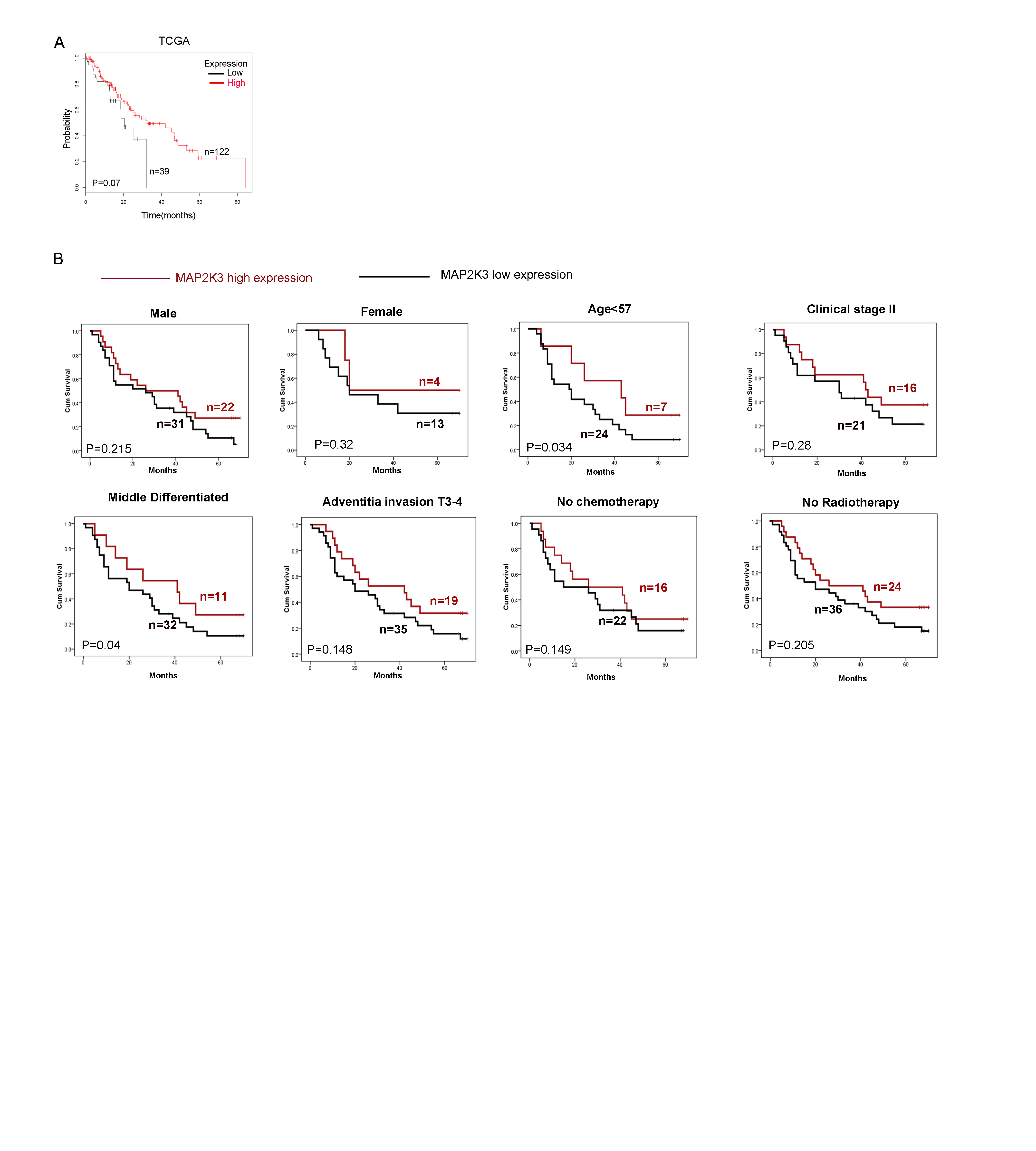

Supplement: Supplementary file 3 — Fig S3. Kaplan‐Meier analysis of MAP2K3 in ESCC patients. [file MOL2-15-1566-s006.tif]

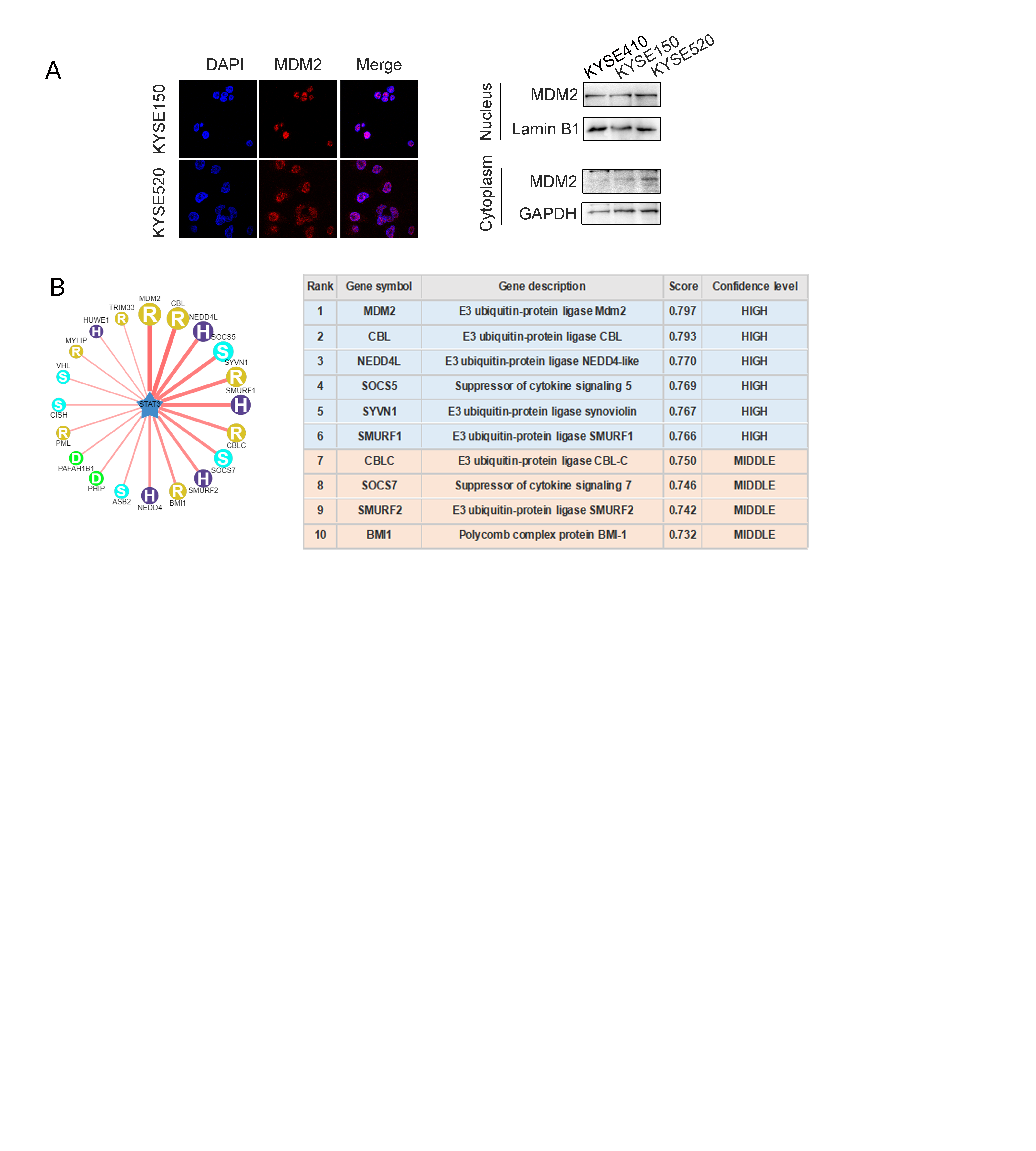

Supplement: Supplementary file 5 — Fig S5. Identification of MDM2 as E3 ligase of STAT3 by bioinformatics. [file MOL2-15-1566-s001.tif]

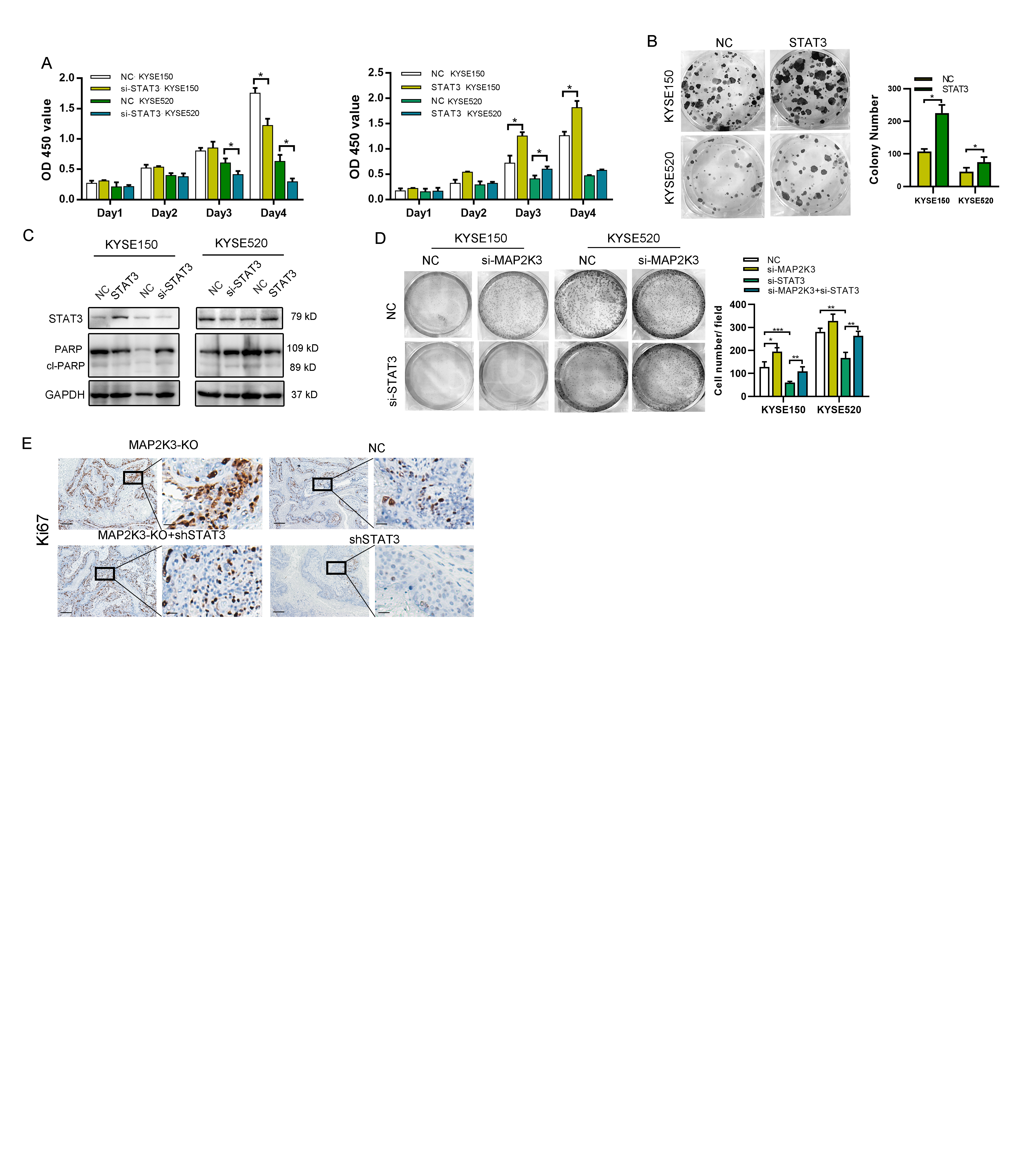

Supplement: Supplementary file 6 — Fig S6. The function of STAT3 in ESCC. [file MOL2-15-1566-s008.tif]

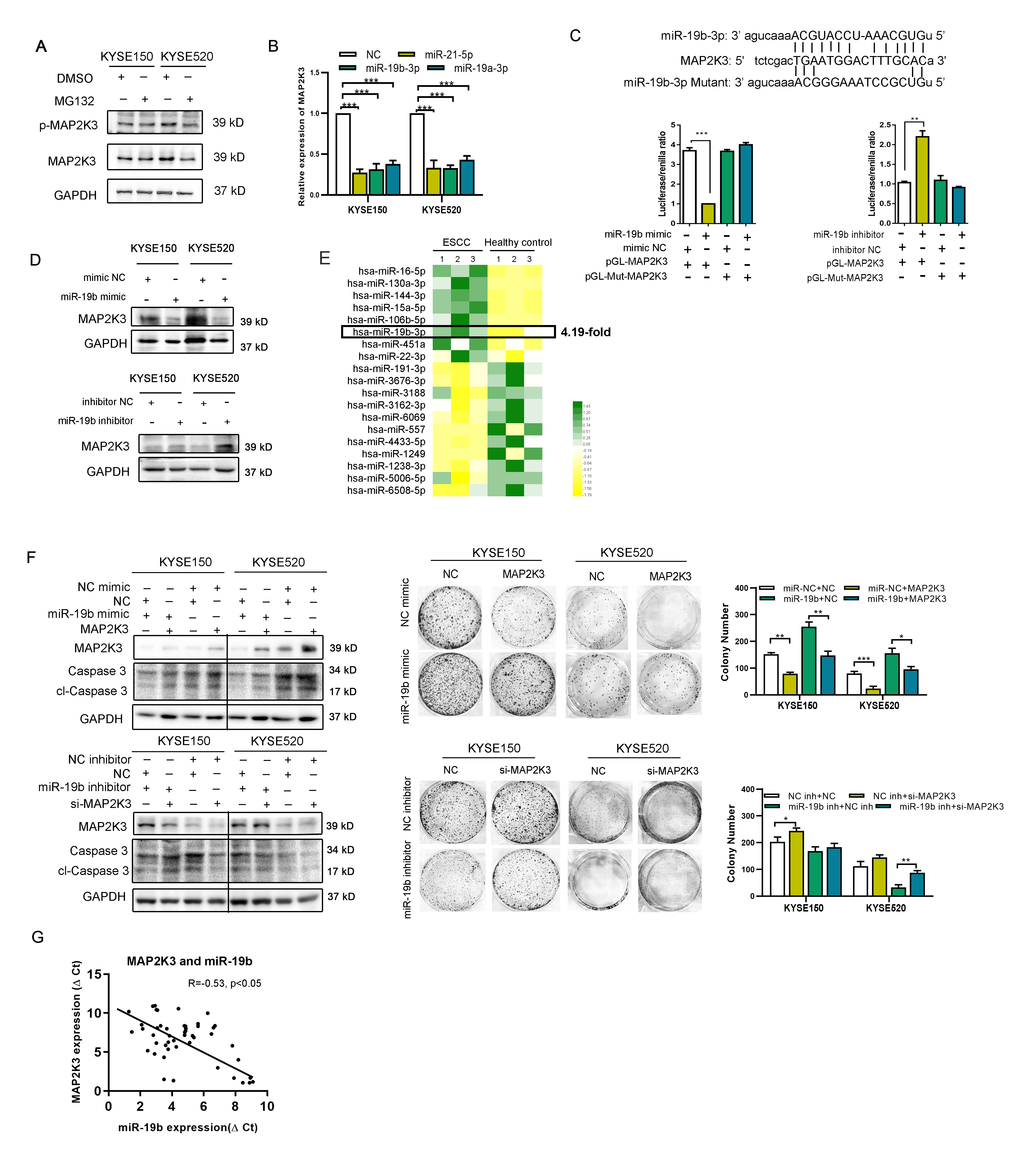

Supplement: Supplementary file 7 — Fig S7. MAP2K3 was suppressed by miR‐19b‐3p in ESCC cells. [file MOL2-15-1566-s009.tif]

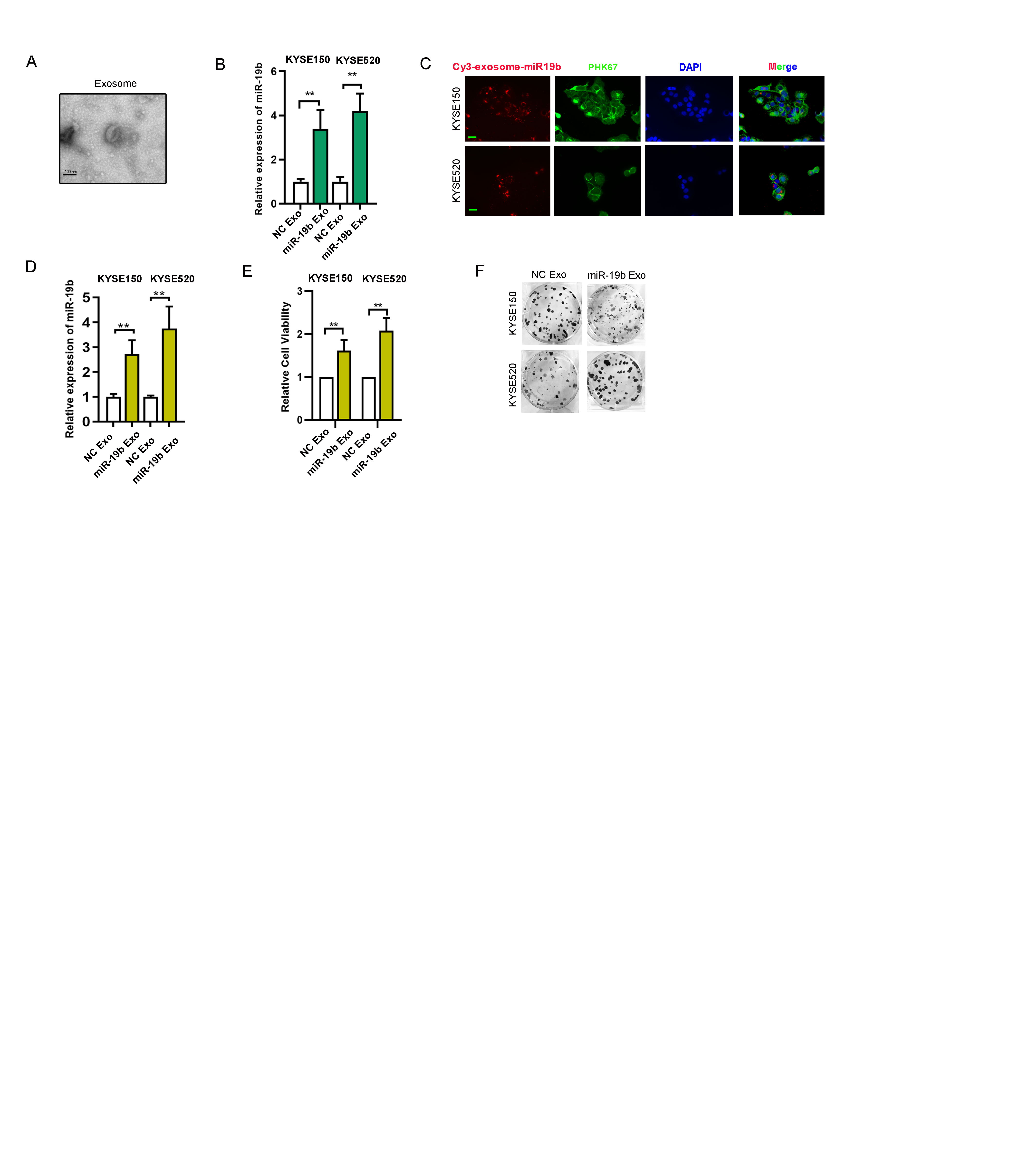

Supplement: Supplementary file 8 — Fig S8. Exosomal miR‐19b‐3p transferred in ESCC cells. [file MOL2-15-1566-s004.tif]
