# Supplementary material for: The miR‐19b‐3p‐MAP2K3‐STAT3 feedback loop regulates cell proliferation and invasion in esophageal squamous cell carcinoma
Source: Mol Oncol. 2021 Mar 14;15(5):1566–83. doi: 10.1002/1878-0261.12934 (PMC8096789; doi:10.1002/1878-0261.12934)
Supplement: Supplementary file 9 — Table S1. CRISPR/CAS9 kinome gene list. [file MOL2-15-1566-s002.docx]

**Supplemental Table 1. CRISPR/CAS9 kinome gene list**

| **Gene name** | ILKAP | PMVK | PXYLP1 | LPIN1 | HIPK3 | DUSP14 | DUSP2 | RIOK3 | PHKA2 |
| --- | --- | --- | --- | --- | --- | --- | --- | --- | --- |
| CRKL | MAPKAPK2 | PGAM1 | FRS2 | PPP2R2C | CDKN3 | GALK2 | SPHK1 | TRIB2 | CAMKV |
| NRBP1 | PPIP5K2 | HYKK | SBF1 | CAMK1 | CMPK2 | RIPK3 | MMD2 | ALPPL2 | TEC |
| PTPN23 | MAPK15 | PIK3R2 | MAP4K1 | PNCK | MAP2K2 | PTPRJ | STK17A | SRP72 | PBK |
| PPP1CB | DGKQ | AK7 | PTPRT | PPP1CC | CSNK1G1 | PDIK1L | PRKCI | PPM1J | NME3 |
| KSR1 | NUDT4 | PPP2R5A | PDXK | BRD4 | PTPRM | PPEF1 | PSKH2 | MUSK | WNK1 |
| EFNA3 | SCYL1 | SOCS3 | CKMT2 | CDC14B | BRAF | DUSP4 | PPFIA2 | ICK | CDC14A |
| GNE | MAP3K1 | BCKDK | UHMK1 | RAF1 | PPP3CB | LMTK2 | ALPK1 | ANKK1 | MELK |
| NADK | TNS3 | PPM1G | NDRG1 | MARK4 | TSKS | CDK11A | GALK1 | CDC42BPA | BRDT |
| PPP1R15B | DGKA | NCK1 | IMPA2 | MAP3K19 | FES | CDK8 | EPHA4 | SGK494 | PRKDC |
| PODXL | OXSM | DDR2 | CILP | NME7 | TIMM50 | EIF2AK4 | PPP1R2 | MTMR3 | NEK2 |
| PTPMT1 | SSH1 | PRKCG | CKB | CIT | PTP4A1 | NTRK1 | INPP1 | G6PC2 | UCK1 |
| PANK3 | PPM1D | DLG3 | PTEN | CAB39L | PPP1R16B | FGR | ERBB2 | MYLK | ACP2 |
| PPM1K | ALPL | PANK1 | PPTC7 | NUDT11 | SYNJ2 | MAP3K6 | PRPS1 | RIPK4 | STYX |
| CCNB1 | DUSP21 | NME8 | PTPRU | PFKFB4 | LPIN2 | GRK1 | TRPM7 | PRKCQ | IP6K1 |
| RPS6KC1 | ALPI | TXK | DLGAP5 | PINK1 | EYA3 | PTPN18 | AURKB | SSH3 | MAGI3 |
| MTMR2 | NRBP2 | HIPK4 | GLYCTK | ITPKC | CHKA | MOS | SBK1 | STK19 | DLG4 |
| DUSP12 | TMEM55B | BCR | PTPRN | PRKCB | PRKCH | PPP1R3C | KHK | INPP5A | HK1 |
| INPP4B | ERLIN2 | TRIB3 | RPS6KL1 | STK32B | NUDT14 | PGK2 | BMX | PIP5K1A | PPP1R7 |
| TGFBR1 | WEE2 | IGBP1 | PIP5KL1 | AGK | AXL | ETNK1 | AK1 | PSKH1 | MAP3K5 |
| ADCK2 | STK25 | STK4 | PTPN12 | BMPR1A | PI4KB | SMG1 | TPTE | FASTK | DMPK |
| MYO3B | PDXP | DBF4 | PHPT1 | PHKA1 | MAP2K5 | CDK1 | PRPS1L1 | MAST2 | INPP5K |
| PIK3R4 | PPP4R1 | DUSP8 | PKN3 | SHC3 | MPP3 | SHPK | NME6 | EPHB6 | PPM1B |
| EYA1 | DUSP11 | MMD | PPP1R12C | TPTE2 | CSNK1G2 | PRKCE | UCK2 | DCK | PIK3C3 |
| CCT2 | YES1 | PRKCD | RNGTT | DOLK | GIT1 | ACVR1 | PRKACG | PTPRR | ADK |
| SSU72 | PPP6C | FZR1 | HUNK | EYA2 | ADCK1 | DUSP15 | NEK8 | GRK5 | NMRK2 |
| COASY | TRIO | EXOSC10 | CSNK2A1 | LMTK3 | PIK3CB | CKM | TNNI3K | CSNK2B | PEAK1 |
| RFK | NEK4 | PIP5K1B | EPHA1 | PI4K2B | PPP5C | PTPRH | GRK4 | MAP3K3 | EPHB2 |
| BMP2K | MAST1 | PTPN9 | DUSP16 | DUSP13 | NT5C1B | MPP2 | WNK4 | MAPK10 | STRADB |
| CERK | PCK2 | GSG2 | PPP2R2D | ATR | AKT1 | GK5 | DGKG | SRMS | STK39 |
| PPM1F | STK26 | IKBKG | TAOK2 | MOK | FN3K | RIOK1 | PTPN11 | NAGK | ACVR1B |
| ILK | SGK2 | NOL9 | NT5C1A | PTPN3 | CNKSR3 | PRKAR2A | TLK2 | PTPRO | DYRK1A |
| STK31 | CRIM1 | WBP11 | STRADA | ATM | PANK4 | LPIN3 | CLK1 | ROR1 | CAMK4 |
| EGFR | THTPA | PPP1R8 | OXSR1 | PIKFYVE | NRP2 | MAPK14 | STK32C | MTM1 | AK9 |
| HKDC1 | CKS1B | SRPK1 | CSNK1A1 | RYK | PFKFB2 | ERN2 | PPP2R2B | TIE1 | MAP3K10 |
| MAPK11 | THNSL1 | RIOK2 | PRKG1 | MPP6 | TNK2 | TTBK2 | DGKI | DAPK2 | EPHA5 |
| PGK1 | DUSP23 | AK8 | CTDP1 | CHKB | PPP3CC | DGKB | STKLD1 | INPP4A | PTPRZ1 |
| PPP2R1A | PTPN7 | PPP1R12A | TSSK6 | DUSP1 | CSNK2A2 | PIK3C2B | NIM1K | CTDSP2 | NUCKS1 |
| DGKH | MAPK6 | EPHA7 | VRK1 | TSSK2 | CAMK2G | TTBK1 | CDC42BPB | ITPKB | GRK7 |
| CAMK2A | GK2 | MYLK4 | PIK3R6 | NMRK1 | G6PC3 | PTPRC | EFNB2 | FXN | SPEG |
| PRPF4B | PPP1R3A | DSTYK | GUK1 | ADPGK | DUSP3 | PRKX | PTPN1 | COL4A3BP | MAP3K14 |
| CTDSPL2 | NUDT1 | PPM1M | CDKL4 | STK40 | MTOR | DLG1 | PALD1 | PI4K2A | CDK6 |
| PTPRE | RPS6KB2 | SEC16A | MVK | IPPK | RPS6KB1 | CDC7 | IRAK2 | IRAK1 | NEK9 |
| MTMR14 | PHKG1 | ACPT | AAK1 | TRIB1 | PIP5K1C | ALPK2 | PAK4 | MAK | PIK3CA |
| MAPK1 | NUAK1 | PON1 | MAP3K7 | TMEM55A | ATRIP | NUDT3 | PRKG2 | PTPN2 | PIM3 |
| AK6 | PGAM2 | MTMR8 | TRPM6 | INSRR | CAMK1D | APTX | MTMR1 | MLKL | MAP3K13 |
| TTK | MTMR9 | FGFR1OP | SKAP1 | XYLB | PTK2 | CDK17 | ACVR2B | PDK2 | OSR1 |
| EFNA2 | CDK11B | HUS1 | GOLGA5 | PPFIBP2 | SGPP2 | CCNB2 | ULK3 | MYO3A | CDK13 |
| PAK2 | DGUOK | LHPP | PDP1 | HSPB8 | BRD3 | MAP3K9 | GUCY2D | FGFR1 | UCKL1 |
| CAMK2N1 | RSPO3 | INPP5J | PLK3 | PTPRB | KDR | NADK2 | STK17B | PDGFRB | PPM1H |
| PDP2 | CDC25A | LCK | NEK11 | NEK5 | EPHB1 | CKS2 | PIK3C2G | PTK7 | RPS6KA6 |
| PPFIA4 | NT5C3B | PRKAB1 | DGKD | SYK | ROR2 | MAP3K11 | ACVRL1 | PPM1E | BRSK2 |
| MAPK3 | INPP5E | NUAK2 | ITPK1 | PDGFRA | TWF1 | FLT1 | NME4 | CDK16 | TKFC |
| EPM2A | CMPK1 | NUDT15 | MAP3K12 | DUSP9 | CAMK2B | GCK | NEK7 | CAMK2D | BRD2 |
| CTDNEP1 | PTPRS | STK11 | PIM2 | ETNK2 | MAST3 | TGFBR2 | DUSP7 | CPNE3 | PDK3 |
| GRB2 | MAP4K5 | GSK3A | NTRK2 | EPHA3 | TESK1 | MINK1 | PTPRA | GSK3B | TSSK1B |
| AK2 | TPK1 | CAMK1G | HK2 | FGFR2 | PTPRN2 | ROS1 | DGKK | PRKAG2 | HCK |
| TP53RK | ABL1 | SET | HIPK2 | PPP1R14B | EEF2K | PDPK1 | PDCD1 | PRKAA2 | PKLR |
| RASSF2 | PRKACB | CDKL1 | CLK4 | WNK2 | PIP4K2A | PAK6 | MAP2K6 | PRKAR1A | CDADC1 |
| NEK1 | CSN2 | CDK2 | ABL2 | FLT3 | STK38 | PPP2CB | IKBKE | MYLK2 | PTPDC1 |
| TYK2 | AURKC | TGFBR3 | JAK1 | MAP4K4 | LIMK2 | CDK5R1 | MAP3K8 | RPS6KA3 | PLK1 |
| HK3 | STK32A | NUDT12 | PIK3R5 | PTPRG | HIPK1 | PDGFRL | MAPKAPK3 | ERBB3 | CCND3 |
| IPMK | BMPR2 | SGPP1 | CLP1 | FRK | PPM1L | CSNK1A1L | TEX14 | SRPK3 | PIP4K2B |
| STK33 | ACP5 | MAPK12 | EPHA2 | PPP1R13B | DYRK3 | TBCK | PPIP5K1 | RET | CDK9 |
| RPS6KA4 | AURKA | PPP1R1C | NEK3 | AK3 | PPP2R3A | PAK1 | EFNA4 | DLG2 | SIK2 |
| PLK2 | GDPD4 | NRK | EIF2AK2 | NUDT2 | ZAP70 | TK2 | MAP4K2 | CDK4 | CDK18 |
| DUSP10 | STC1 | MTMR7 | MAP2K1 | SPHK2 | PPP1CA | PTK2B | NME2 | PIK3R1 | MAP3K4 |
| PRKAR1B | LTK | PHKB | IRAK3 | PRKAB2 | ERBB4 | STK16 | DUSP6 | PRKCA | DAPK1 |
| ADCK5 | DTYMK | PCK1 | PDK4 | IP6K3 | CDKL5 | LRRK1 | FGFR1OP2 | PPP3CA | JAK2 |
| VRK2 | DCLK2 | CSNK1D | ACVR1C | TSSK4 | INPPL1 | LRRK2 | ALDH18A1 | PTPN13 | DUSP27 |
| ALPP | MYLK3 | SLK | DGKE | EIF2AK1 | PIK3AP1 | SSH2 | BPNT1 | PIK3C2A | SACM1L |
| DCLK1 | NUDT9 | MAPK7 | AK5 | ACP1 | SRC | EPHA8 | PIM1 | ACPP | MAP2K3 |
| TNIK | CARD11 | PKMYT1 | IRAK4 | PGAM4 | AMHR2 | GK | NT5C3A | MARK2 | MAP2K7 |
| PPP2R5D | MET | SIRPA | KSR2 | VRK3 | DOK1 | MTMR4 | PFKFB1 | PTPN6 | GAB1 |
| PAK3 | ERN1 | RPS6KA2 | MAGI1 | BMPR1B | CDK12 | ROCK1 | ULK2 | PPFIA3 | EFNA5 |
| NT5C | UBLCP1 | CDK3 | CSF1R | PPP1R1A | CDKL2 | CDK10 | SIK3 | CHUK | CHEK1 |
| PKDCC | AKT2 | ROPN1L | WNK3 | TAOK1 | ROCK2 | CDK15 | PSPH | RPS6KA5 | CDK7 |
| STYXL1 | DCLK3 | TAOK3 | EPHB3 | TRAT1 | PRKD1 | PPP3R2 | TESK2 | MAP4K3 |  |
| DYRK2 | PNKP | AK4 | CIB2 | MST1R | GRK6 | PACSIN1 | CDK20 | PTPN4 |  |
| PPP2R5B | PFKM | TSSK3 | ALPK3 | BRSK1 | FN3KRP | PRKAR2B | BUB1B | INSR |  |
| LIMK1 | FUK | NME1 | FBP2 | ACP6 | NCK2 | ULK1 | PTPN22 | STK24 |  |
| STK35 | NEK6 | PFKP | LATS1 | ITK | CDK14 | MPP4 | TBK1 | CDC25B |  |
| PTPN14 | PPEF2 | MKNK1 | TEK | SRPK2 | PIP4K2C | CCNC | FBP1 | PTPN5 |  |
| TWF2 | PRKD3 | INPP5B | SYNJ1 | OCRL | DAPK3 | MAPK9 | ITPKA | PRPS2 |  |
| SNRK | PLK4 | PTPN20 | CAMKK2 | GUCY2C | PPP4C | ZMYM2 | STK38L | TYRO3 |  |
| PEG3 | MTMR6 | LYN | NLK | PPP2R1B | RIPK2 | DUSP5 | FGFR3 | PPP2R2A |  |
| MPP1 | GAK | BUB1 | MPP5 | CSK | AATK | DYRK1B | PRKACA | PRKCZ |  |
| CSNK1E | TLK1 | EYA4 | ACVR2A | PPP1R1B | PANK2 | PTPRF | MINPP1 | PTPRD |  |
| BLK | PXK | OBSCN | BAIAP2 | PPP2R5E | PKM | IKBKB | MERTK | CDKN1B |  |
| CAMKK1 | MDP1 | MAPK8 | DOT1L | PTP4A2 | CTDSP1 | GUCY2F | PTP4A3 | ARAF |  |
| CASK | NUDT5 | DUSP18 | TMEM134 | MARK1 | DDR1 | TTN | MAPK4 | WEE1 |  |
| PPP3R1 | PKN2 | MAP2K4 | CDC37 | FYN | MASTL | LATS2 | CDC25C | TNK1 |  |
| PTPN21 | NT5M | STYK1 | MKNK2 | HACD2 | TJP2 | NPRL2 | SGK1 | CDKN1A |  |
| MAGI2 | CDC42BPG | DUSP19 | MAPK13 | RPS6KA1 | CDK19 | PFKL | MARK3 | STK3 |  |
| CHEK2 | CSNK1G3 | PKN1 | BTK | ULK4 | PTPRK | CDK5 | CCNK | PIK3CD |  |
| PPM1A | EFNB3 | PI4KA | PELI2 | MEX3B | EIF2AK3 | PPP1R3D | PIK3R3 | BPGM |  |
| NT5E | PSTK | TRMT2A | PTK6 | PHKG2 | SCYL3 | NT5C2 | IMPA1 | POMK |  |
| EPHB4 | MATK | PRKD2 | PPP2CA | RBKS | DUSP22 | JAK3 | CDKL3 | PRKAA1 |  |
| ALK | TK1 | G6PC | RIPK1 | NME5 | INPP5D | DYRK4 | PDK1 | PFKFB3 |  |
| MAP3K2 | CLK3 | AKT3 | CLK2 | STK36 | STK10 | PPP1R14A | PRKAG1 | NTRK3 |  |

**Supplemental Table 2.** **Primers**

| **STAT3** | 5'-GGAGCAGAGATGTGGGAATG-3' | 5'-CTTGGTGGTGGAGGAGAACT-3' |
| --- | --- | --- |
| **MAP2K3** | 5’-GACTCCCGGACCTTCATCAC-3’ | 5’-GGCCCAGTTCTGAGATGGT-3’ |
| **STAT1** | 5'-CAGCTTGACTCAAAATTCCTGGA-3' | 5'-TGAAGATTACGCTTGCTTTTCCT-3' |
| **Cyclin D1** | 5'-GGATGCTGGAGGTCTGCGA -3' | 5'-TAGAGGCCACGAACATGCAAG-3' |
| **Survivin** | 5'-CAAGGAGCTGGAAGGCTGG-3' | 5'-GTTCTTGGCTCTTTCTCTGTCC-3' |
| **VEGF** | 5’-AAAGGAGCCTACAAGA-3' | 5'-TTCACAAGCAGCCAAT-3' |
| **β-actin** | 5'-AGCAAGCAGGAGTATGACG-3' | 5'-GTGGGGTGGCTTTTAGGA-3' |
| **miR-19b-3p** | 5'-CGAGCCGGATCCGTTAG-3' | 5'-CGACTAGGCTTCGCTAGA-3' |
| **STAT3 binding Primer 1** | 5’-TCCAGTCATACACGTGGACC-3’ | 5’-ACCCATTCCAGAAAACTTCCTT-3’ |
| **STAT3 binding Primer 2** | 5’-AAGGAAGTTTTCTGGAATGGGT-3’ | 5’-AACTGCATAAGCCAGTTTCCAA-3’ |
| **U6** | 5'-CGCTTCGGCAGGCATTATATAC-3' | 5'-AAGGGGCCATGCTAATCTT-3’ |
