# Supplementary material for: The miR‐19b‐3p‐MAP2K3‐STAT3 feedback loop regulates cell proliferation and invasion in esophageal squamous cell carcinoma
Source: Mol Oncol. 2021 Mar 14;15(5):1566–83. doi: 10.1002/1878-0261.12934 (PMC8096789; doi:10.1002/1878-0261.12934)
Supplement: Supplementary file 10 — Table S2. Primers. [file MOL2-15-1566-s010.docx]

| STAT3 | 5'-GGAGCAGAGATGTGGGAATG-3' | 5'-CTTGGTGGTGGAGGAGAACT-3' |
| --- | --- | --- |
| MAP2K3 | 5’-GACTCCCGGACCTTCATCAC-3’ | 5’-GGCCCAGTTCTGAGATGGT-3’ |
| STAT1 | 5'-CAGCTTGACTCAAAATTCCTGGA-3' | 5'-TGAAGATTACGCTTGCTTTTCCT-3' |
| Cyclin D1 | 5'-GGATGCTGGAGGTCTGCGA -3' | 5'-TAGAGGCCACGAACATGCAAG-3' |
| Survivin | 5'-CAAGGAGCTGGAAGGCTGG-3' | 5'-GTTCTTGGCTCTTTCTCTGTCC-3' |
| VEGF | 5’-AAAGGAGCCTACAAGA-3' | 5'-TTCACAAGCAGCCAAT-3' |
| β-actin | 5'-AGCAAGCAGGAGTATGACG-3' | 5'-GTGGGGTGGCTTTTAGGA-3' |
| miR-19b-3p | 5'-CGAGCCGGATCCGTTAG-3' | 5'-CGACTAGGCTTCGCTAGA-3' |
| STAT3 binding Primer 1 | 5’-TCCAGTCATACACGTGGACC-3’ | 5’-ACCCATTCCAGAAAACTTCCTT-3’ |
| STAT3 binding Primer 2 | 5’-AAGGAAGTTTTCTGGAATGGGT-3’ | 5’-AACTGCATAAGCCAGTTTCCAA-3’ |
| U6 | 5'-CGCTTCGGCAGGCATTATATAC-3' | 5'-AAGGGGCCATGCTAATCTT-3’ |

**Supplemental Table 2.** **Primers**
